# *Buyang Huanwu* decoction affects gut microbiota and lipid metabolism in a ZDF rat model of co-morbid type 2 diabetes mellitus and obesity: An integrated metabolomics analysis

**DOI:** 10.3389/fchem.2022.1036380

**Published:** 2022-11-09

**Authors:** Mei Liu, Qinmian Zhao, Jiayan Liu, Aijing Huang, XinHua Xia

**Affiliations:** ^1^ School of Agriculture and Biology, Zhongkai University of Agriculture and Engineering, Guangzhou, China; ^2^ The First Affiliated Hospital of Guangzhou Medical University, Guangzhou, China; ^3^ Institute of Integrated Chinese and Western Medicine, Guangzhou Medical University, Guangzhou, China

**Keywords:** Buyang Huanwu decoction, type 2 diabetes mellitus, obesity, glucose and lipid metabolism, lipid metabolomics

## Abstract

Type 2 diabetes mellitus (T2DM) is a chronic disease associated with many severe complications such as blindness, amputation, renal failure, and cardiovascular disease. Currently, the prevention and treatment of T2DM is a major global challenge as the number of aging and obese people is increasing. Traditional Chinese medicine offers the advantages of multi-target holistic and individual treatment for obesity and type 2 diabetes. However, most of the TCMs for T2DM are not scientifically evaluated. Here, *Buyang Huanwu* decoction (BYHWD), a widely used TCM formula, was used to explore scientific pharmacological activity against T2DM in rat models. First, BYHWD exhibited excellent inhibitory actions against body fat accumulation and increased blood triglyceride levels, and a high-fat diet (HFD) induced blood glucose elevation in diabetic rats. Moreover, 16S rDNA sequencing of fecal samples identified the distinct changes in the community composition of gut flora following BYHWD treatment, displayed as significantly increased *Bacteroidetes* and dramatically decreased *Firmicutes* at the phyla level, and the remarkable increase in the abundance of *Lactobacillus* and *Blautia*. Additionally, lipid metabolomics based on liquid chromatography–mass spectrometry revealed a significant shift of lipid metabolites in the liver after BYHWD treatment. Notably, these differential lipid metabolites were particularly involved in biological processes such as cholesterol metabolism, linoleic acid metabolism, glycerolipid metabolism, glycerophospholipid metabolism, insulin resistance, arachidonic acid metabolism, and alpha-linoleic acid metabolism. Importantly, Spearman correlation analyses suggested an association between disturbed gut microbiota and altered lipid metabolites. Moreover, they were also closely associated with the bioactivities of BYHWD to reduce the blood lipid and blood glucose levels. Collectively, these results suggest that BYHWD could meliorate gut microbiota dysbiosis and lipid metabolite alterations induced by the HFD in diabetic rats. These results not only provide a novel perspective on understanding the mechanisms underlying BYHWD bioactivity against T2DM but also suggest the use of advanced systems biology methods to reveal some unknown scientific laws in TCM theories.

## 1 Introduction

Diabetes is a metabolic disease, with type 2 diabetes (T2DM) accounting for the largest proportion (i.e., almost 90% of all diabetes) (Kahn et al., 2006). T2DM has a complex etiology and is closely linked to aging and environmental and lifestyle changes (Brody, 2012). The discovery of insulin transformed T2DM from a fatal disease into a preventable, controllable, and treatable one. Over the past 100 years, the updation and iteration of different insulin types and the emergence and application of new hypoglycemic drugs such as GLP-1 receptor agonists have significantly improved the awareness, diagnosis, treatment, and control rate of T2DM. However, the overall prevention and treatment status of diabetes remains unoptimistic. First, T2DM has a long course and complex condition, which is susceptible to many complications, such as blindness, amputation, renal failure, and cardiovascular disease. These complications severely affect patient health, eventually leading to death. Second, as the population is aging, the overall prevalence of T2DM continues to increase because of a high incidence of obesity and other factors. Thus, T2DM has become a global challenge (Després and Lemieux, 2006). Obesity is known as the most crucial risk factor for diabetes, and China alone has more than 180 million obese people (Wang et al., 2021). Thus, the prevention and treatment of T2DM is still beyond the horizon. The discovery of more effective therapies or drugs to prevent or treat T2DM and its associated complications remains a major demand for medical development globally.

Obesity is the precursor and inducer of T2DM. Modern medical research defines obesity as abnormal or excessive fat accumulation that poses a threat to public health and has been described as one of the most evident but easily overlooked health concerns of today (Partridge et al., 2018). Evidence has proved that obesity results from the imbalance between energy intake and expenditure. The gut is a vital part of the digestive system and a crucial source of energy metabolism. Hence, the gut microbiota is an important environmental factor which can lead to obesity by changing the host’s energy acquisition and storage capacity (Qin et al., 2012; Tremaroli and Bäckhed, 2012; Pedersen et al., 2016). For example, some studies have revealed that bacterial diversity and gene richness in obese individuals are lower than those in lean individuals. Moreover, the obese individuals exhibited a low bacterial richness characterized by more marked overall adiposity, insulin resistance, dyslipidemia, and a more pronounced inflammatory phenotype than the lean individuals with a high bacterial richness (Cotillard et al., 2013; Le Chatelier et al., 2013). Meanwhile, intestinal microbiota has been reported to change easily in response to external influences. Some experimental studies on intestinal microbiota transplantation have shown a causal relationship between intestinal microbiota and obesity development, which can link the dysregulation of intestinal microbiota with obesity. For instance, a study found that adult germ-free mice did not become obese after feeding on a high-fat diet (HFD) (Ridaura et al., 2013). Other studies have also reported that germ-free mice transplanted with gut microbiota from normal, obese, and lean mice eventually exhibited no significant changes in body weight, obesity, and wasting, respectively (Rabot et al., 2010). Because the gut microbiota has the most important role in treating obesity or obesity-linked diseases, the intestinal flora has become a new target for obesity control and intervention for obese T2DM patients (Forslund et al., 2015).

Traditional Chinese medicine (TCM) embodies 5,000 years of medical philosophy and practical experience of the Chinese nation. It offers the advantages of multi-target holistic treatment and individual treatment in the intervention of obesity and T2DM. Therefore, it may be a feasible and necessary approach to find new therapies or drugs that can allow effective early prevention and treatment of T2DM. A widely used formula in TCM, *Buyang Huanwu* decoction (BYHWD) has been proven to simultaneously exhibit a considerable therapeutic effect on the regulation of lipid and glucose metabolism disorders in clinical practice. This popular formula is composed of seven herbal medicines. Among them, Radix Astragali is the key herbal medicine, with its amount in the BYHWD formula being five-fold higher than the total amount of the other six herbal medicines. Notably, Radix Astragali is a major ingredient in six of the seven antidiabetic herb formulas approved by the State Drug Administration of China (Xu et al., 2009). However, the specific bioactivity and possible action mechanism of BYHWD against T2DM remain unclear. Thus, the present study explored the impacts of BYHWD on the intestinal flora and liver lipid metabolism and their association with the effects of BYHWD on blood sugar regulation and reduction in obesity in an obese T2DM rat model. Our investigation of the BYHWD-mediated regulation of glucose and lipid metabolism in obese diabetic rats may help identify targeted TCM-based therapies to comprehensively treat and/or prevent T2DM.

## 2 Material and methods

### 2.1 Drugs and reagents

BYHWD is composed of seven different traditional herbal medicines. Their names and proportions are given as follows: Astragali Radix (120 g), Angelicae Sinensis Radix (6 g), Paeoniae Rubra Radix (4.5 g), Chuanxiong Rhizoma (3 g), Persicae Semen (3 g), Carthami Flos (3 g), and Pheretima (3 g). All herbal medicines used in the present study were acquired from Kangmei Pharmaceutical Co., Ltd. (Guangzhou, China). These herbal medicines were processed from the respective raw plants under strict quality control measures in accordance with the China Pharmacopeia 2020 by the manufacturing company. The voucher specimens of these herbal medicines were deposited at the Chinese Medicine Specimen Center at Guangzhou Medical University.

The BYHWD extract used in the present study was prepared in our laboratory. Briefly, 10 doses of the original BYHWD were uniformly mixed, and the mixture was soaked in distilled water for 1 h and then boiled twice. During the first boiling step, the volume of water was maintained at 10-folds of the medicinal material, and the boiling time was set to 1.5 h. In the second boiling step, the boiled water volume was maintained at eight-folds of the dry ingredients, and the boiling time was set to 1 h. After two boils or extractions, the water mixture was filtered through a #4 filtration paper, and the filtrate was evaporated under reduced pressure rotary and condensed to the extract with a density of 1.2–1.4. Subsequently, the thick extract was placed in a vacuum freezer for freeze drying, and the solid extract was smashed into a powder form using a 40 mesh and was stored in a −20°C refrigerator.

The metformin hydrochloride tablet (DMBG) obtained from Sino-US Shanghai Shi Guibao Co., Ltd. was used as the positive control drug. H&E and Oil Red O staining kits were purchased from Servicebio^®^ of Wuhan Servicebio Technology Co., Ltd. The remaining agents used in the present study were all of analytical grade and obtained from Sinopharm Chemical Reagent Co., Ltd.

### 2.2 Animals and treatments

Male Zucker diabetic fatty (ZDF) rats and Zucker lean control (ZLC) rats aged 7 weeks and of specific pathogen-free (SPF) grade were purchased from Charles River Co., Ltd. (Beijing, China; license number: SCXK 2016-0006). All rats were placed in individually ventilated cages under SPF conditions with a constant temperature of 20°C–25°C and humidity of 65%–70%, with a 12-h light/dark cycle, along with free access to food and water. All animal care and experimental protocols were reviewed and approved by the Experimental Animal Ethics Committee of Guangzhou Medical University (GMU-20200018) and followed the guidelines of the Animal Use and Care of the National Institutes of Health (NIH) and Animal Research: Reporting *In Vivo* Experiments (ARRIVE).

Six ZLC rats were assigned to the normal control group, and 28 ZDF rats were used to establish the obese T2DM model. After a week of adaptive feeding, both ZLC and ZDF rats were fed a high-fat diet (HFD) of Purina #5008 (composed of 26.85% protein, 16.71% fat, and 56.44% carbohydrate) for 4 weeks. The random blood glucose level (RBG) of 24 ZDF rats was >16.7 mmol•L^−1^ at the end of 4 weeks’ HFD feeding, which indicated that diabetes was successfully established in these 24 ZDF rats. In contrast, the RBG of ZLC rats remained normal. Then, the ZLC rats were assigned to the control group (*n* = 6), and 24 diabetic ZDF rats were categorized into three groups of eight each: diabetes model group (DM), diabetes with BYHWD treatment (DM + BYHWD), and diabetes with the positive drug group of DMBG treatment (DM + DMBG). Rats from the control and DM groups were provided with double-distilled water; the DM + BYHWD rats were administered the BYHWD extract, and the DM + DMBG rats were administered DMBG *via* the intragastric route. All treatments were processed for 7 weeks, and all rats were continuously fed an HFD during the entire experimental period. The dosage of BYHWD and DMBG was equivalent to the dose for an adult in clinical practice, which is 0.783 g/kg/day of the BYHWD extract and 0.1 g/kg/day of DMBG. The rat-equivalent dose was calculated to be seven times that of a human weighing 70 kg. The BYHWD extract dosage was one dose of the formula taken by an adult in a day. In the present study, a total of 10 dosages of the formula were used in the extraction of BYHWD, and the final weight of the dry extract powder was 78.3 g, which is a 10-day dosage for an adult. Therefore, the equivalent dose of the BYHWD extracts for rats was 7.83 g/kg/day. According to our past unpublished studies, the pharmacological effect of an equivalent dose of BYHWD extracts on rats indicated better therapeutic effect on SD rats with diabetes induced by STZ. Therefore, in the present study, an equivalent dose (0.783 g/kg/day) of an adult was used as the treatment dose for ZDF rats in the DM + BYHWD group. Moreover, 0.5% sodium carboxymethyl cellulose aqueous solution was used to prepare 0.0783 g/ml suspension with a volume of 10 ml/kg for daily administration, and the dosage was calculated according to the body weight of the animal. The equivalent metformin dosage in rats was also an equivalent dose with the clinical dose of 1.0 g/70 kg × 7 that of an adult. Hence, the dose of metformin used for the treatment of DM + DMBG rats was 0.1 g/kg/day. Prior to its daily administration, a 0.5% sodium carboxymethyl cellulose aqueous solution was prepared with 0.01 g/ml metformin solution at the volume of 10 ml/kg, and the dosage was calculated according to the body weight of the animal.

The general status of the rats, including their weight, food, and water intake, was recorded every 2 days since the beginning of HFD consumption. After 4 weeks of model establishment and 7 weeks of drug treatment, all rats were anesthetized and their blood samples were collected *via* the abdominal aorta. In addition, the serum FBG, TG, CHOL, and LDL levels were determined using an automatic biochemical analyzer (Hitachi, Beijing, China). Following blood collection, the rats were sacrificed *via* cervical dislocation; the ligaments and blood vessels surrounding the liver were separated; and the intact liver was immediately and completely removed to weigh and record it for the calculation of the hepatobody ratio (liver weight/body weight), followed by the separation of the pancreatic head, rinsed in pre-cooled PBS and dried with a clean filtration paper for histological examinations. In addition, the perirenal, epididymal, and mesenteric fats were isolated from the related portions of the rats; they were weighed and recorded to calculate the total fat weight/body weight ratio. Finally, the whole cecum was dissected and cut open immediately, and the feces in the cecum were removed and placed into an anaerobic bag and flash frozen in liquid nitrogen. The obtained samples were then frozen at −80°C for 16S rDNA gut microbiota sequencing. All other organ samples were flash frozen in liquid nitrogen immediately after their harvest and stored at −80°C until further analyses.

### 2.3 Histopathological examination of the liver and pancreatic head

Histopathological examinations of the liver and pancreatic head were performed by hematoxylin–eosin (H&E) and Oil Red O dying. H&E dying was conducted as follows: the liver and pancreas were immersed in 4% paraformaldehyde and fixed for >24 h. Next, these tissues were embedded in paraffin and cut into 4-μm slices. All slices were hydrated and stained with hematoxylin–eosin (H&E) solution as per the standard procedure, and the images were subsequently captured using an inverted microscope (Olympus, Toyoko, Japan).

Oil Red O dying was performed as follows: the fresh liver tissues were cut into 8–10-µm frozen sections. After re-warming and drying, the frozen slices were fixed in 4% paraformaldehyde for 15 min, washed in running pure water, and dried. Immediately afterward, the slices were placed into an Oil Red O staining solution for 8–10 min in the dark. Subsequently, the slices were removed from the staining solution and placed in 60% isopropanol for 3 s to initiate differentiation. Next, the slices were re-stained with hematoxylin for 3–5 min and then stained with Evans blue fluid to return it to blue color for 1 s. Finally, the slices were gently immersed in two jars of tap water for 5 s and 10 s, respectively, and then sealed using a glycerin gelatin tablet. Finally, the hepatic lipid accumulation was studied under a light microscope (Olympus).

### 2.4 Sample collection and preparation

All fecal samples were subjected to fecal bacterial DNA extraction with the QIAamp DNA stool MiniKit (Qiagen, Hilden, Germany), according to the manufacturer’s instructions. The NanoDrop 2000 spectrophotometer (Thermo Scientific, Wilmington, United States) and gel electrophoresis with 1% agarose were performed to assess the concentration and purity of the DNA extraction, respectively.

A part of the frozen liver tissue samples was obtained to extract the lipids for lipid metabolomics analysis. Briefly, 50 mg of the frozen liver tissues were accurately weighed and homogenized on an ice bath using the T-18 Ultra-Turrax homogenizer (Vernon Hills, IL, United States). Following centrifugation at 12,000 rpm for 5 min at 4°C, 100 µL of the supernatant was collected and transferred into a 2 ml-EP tube and immediately introduced to a pre-chilled (−20°C) chloroform and methanol mixture (750 μL, 2:1 ratio), followed by vortexing for 30 s. The mixture was then placed in an ice bath for the next 40 min. Subsequently, 190 µL of ddH_2_O was added to the mixture, followed by a 30-s vortex and incubation on an ice bath for an additional 10 min. The mixture was next centrifuged at 12,000 rpm for 5 min at 4°C, and 300 µL of the lower layer fluid was collected and transferred into a fresh 2-ml EP tube. Then, 500 µL of a pre-cooled mixture of chloroform and methanol (2:1) was re-introduced to the collected layer, followed by vortexing for 30 s. The mixture was then centrifuged at 12,000 rpm for 5 min at 4°C. Next, 400 µL of the lower layer fluid was collected again, transferred into a new 2-ml EP tube, and concentrated to form a dry solid in a vacuum concentrator. The dry sample was then dissolved in 200 μL of isopropanol and centrifuged. The supernatant was filtered through a 0.22-µm membrane to obtain the samples for HPLC-MS analysis. Finally, 20 µL of the sample was analyzed for quality control (QC).

### 2.5 16S rRNA sequencing and sequencing data analysis

After extracting the genomic DNA, the primer sequences 515F/806R (515F:5’-GTGCCAGCMGCCGCGGTAA-3’, 806R:5’-GGACTACHVGGGTWTCTAAT-3’) were used for PCR amplifications to augment the V3 plus V4 hypervariable region of the 16S rDNA. PCR amplifications were performed as follows: initial denaturation at 98°C for 30 s was applied, followed by 35 cycles (98°C for 10 s, 54°C for 30 s, and 72°C for 45 s). The gradient L96G PCR instrument (LongGene, Hangzhou, China) was used to qualify the PCR amplifications. Next, the purified amplification was performed in equal amounts and sequenced to construct a sequencing library with the HiSeq2500 PE250 platform (Illumina Inc., San Diego, CA, United States) for paired-end reads.

The paired-end reads were then assembled and merged using Flash software (version 1.2.7) based on the criteria of overlap length ≥10 bp and mismatch proportion ≤10%. Low-quality sequences were filtered out by Microbial Ecology software, and high-quality sequences were clustered into operational taxonomic units (OTUs) using Usearch in QIIME software (version 7.1) based on a 97% sequence similarity to calculate the abundance of OTUs. Maffi software (V7.310) was applied for multiple-sequence alignment to analyze the differential distribution of dominant species across different populations and to study the developmental relationship of different OTUs. In addition, alpha-diversity, including the indexes of Chao1, Simpson, and Shannon, was calculated to evaluate the richness and diversity based on the OTUs among the different samples. Meanwhile, beta-diversity was analyzed by principal component analysis (PCA), principal coordinate analysis (PCoA), and non-metric multidimensional scaling (NMDS) analysis, according to the taxonomic classification and OTU abundance among the samples. Difference comparison of the microbial community abundance on different groups at the genus and phylum levels were conducted by statistical analyses with a significant difference of *p* < 0.05. The LDA effect size (LefSe) analysis about 16S rDNA sequencing was applied with reference to http://huttenhower.sph.harvard.edu/galaxy/.

### 2.6 LC-MS analysis for lipid metabolomics analysis

Chromatographic separation was performed in the Thermo Vanquish System equipped with the ACQUITY UPLC^®^ BEH C18 column (100 × 2.1 mm, 1.7 µm, Waters). The column was maintained at 50°C, and all samples were placed in a sequence in an autosampler at 8°C. The mobile phase was composed of A and B, wherein A represents the combination of acetonitrile and water in a ratio of 60:40 and B represents the combination of isopropanol and acetonitrile in a ratio of 90:10. In addition, 0.1% formic acid was added to acetonitrile, and 10 mM ammonium formate was added to water and isopropanol, respectively. The flow rate of the mobile phase was set to 0.25 ml/min. Then, 2 μL of each sample was injected automatically into the system after system equilibration. An increasing linear gradient of mobile phase A (v/v) was applied to flush the column as follows: 0–5 min, 70%–57% A; 5–5.1 min, 57%–50% A; 5.1–14 min, 50%–30% A; 14–14.1 min, 30% A; 14.1–21 min, 30%–1% A; 21–24 min, 1% A; 24–24.1 min, 1%–70% A; and 24.1–28 min, 70% A.

The ESI-MS^n^ analyses were performed on the Thermo Q Exactive Focus mass spectrometer (Orbitrap) with a spray voltage of 3.5 kV and −2.5 kV in the positive and negative modes, respectively. Sheath and auxiliary gases were set to 30 and 10 arbitrary units, respectively. The capillary temperature was set to 325°C. The Orbitrap analyzer scanned a mass range of 150–2,000 m/z for a full scan at a mass resolution of 35,000. Data-dependent acquisition (DDA) MS/MS analyses were performed using the HCD scan. The normalized collision energy was 30 eV. Dynamic exclusion was implemented to remove the unnecessary information from the MS/MS spectra.

### 2.7 Lipid metabolomics data processing and analysis

Positive- and negative-ion modes were simultaneously applied to collect the data to obtain more comprehensive and complete lipid information from the samples. Lipid Search software (V4.2) was used to sequentially annotate the collected raw data (*.raw format). Next, the data matrix was established, which included the mass-to-charge ratio (M/Z), retention time (RT), and intensity. The annotation results of all samples were aligned using Lipid Search software (V4.2), and the peak alignment and peak filtering were performed on the annotation results of both positive- and negative-ion modes of all single samples. The main parameters were set to 0.25 of RT tolerance and 5 of M-score threshold. The high-peak response values in the test samples and the low RSD values in the QC samples were retained for samples with both positive- and negative-ion modes. In addition, total peak normalization of the peak response values was performed for all raw data in order to enable the comparison of data with differing magnitudes.

### 2.8 Statistical analyses

All statistical results were expressed as mean ± standard deviation (X ± S.D). The experimental data were analyzed using SPSS software (version 25.0, SPSS Inc., Chicago, United States) and GraphPad Prism (version 9.0, GraphPad Software Inc., California, United States). One-way ANOVA was applied for an independent sample comparison, whereas non-normal distribution data were tested by the non-parametric test method. For inter-group comparisons, the LSD method was used when the homogeneity of variance was matched, and the non-parametric test was applied when the homogeneity of variance was unmatched. Pairwise correlations between the pharmacological factors, gut microbiota, and lipid metabolites were conducted by Spearman correlations, and the correlograms were generated by R software (version 4.0.5). The *p*-value was used to represent statistical significance. When *p* < 0.05, a significant difference was considered, and when *p* < 0.01, the significant difference was considered to be enormous.

## 3 Results

### 3.1 Identification of components from the BYHWD extract using LC-MS

The water extracts of BYHWD were prepared as described in methods in Section 2.1. Before ingestion, potential active components in these extracts were examined using LC-MS, according to the accurate molecular weight matching with components in the data matrix. As displayed in Supplementary Figure S1A, 77 components were identified in the water extract. Among them, more than half of all identified components were isolated from Radix Astragali (Supplementary Table S1), which contributed 35 components to the BYHWD extract. These components were mostly composed of typical Astragalus saponins such as astragaloside I, astragaloside II, astragaloside III, astragaloside IV, and astragaloside V (Supplementary Figures S1B, S1C). Paeoniae Rubra Radix contributed the second largest number of the identified components in the BYHWD extract. In total, 20 ingredients were associated with Paeoniae Rubra Radix including oxypaeoniflora, albiflorin, albiflorin R1, and benzoyloxypaeoniflorin. Of the remaining identified components, seven were from Angelicae Sinensis Radix. Most of these components were organic acids, including ferulic acid, which is a principal component of Angelicae Sinensis Radix. Chuanxiong Rhizoma contributed five ingredients, which comprised characteristic components such as Z-ligustilide and ligustilide dimer. Moreover, five components were from Persicae Semen, including prunasin. Furthermore, although 72 components in BYHWD were from major herbal plants, only four components were identified from Carthami Flos and one component from Pheretima. This was attributable to the relatively small proportion of both herbs in the BYHWD water extract.

### 3.2 *Buyang Huanwu* decoction prevents high-fat diet-stimulated weight increase and fat accumulation in diabetic ZDF rats

**FIGURE 1:**
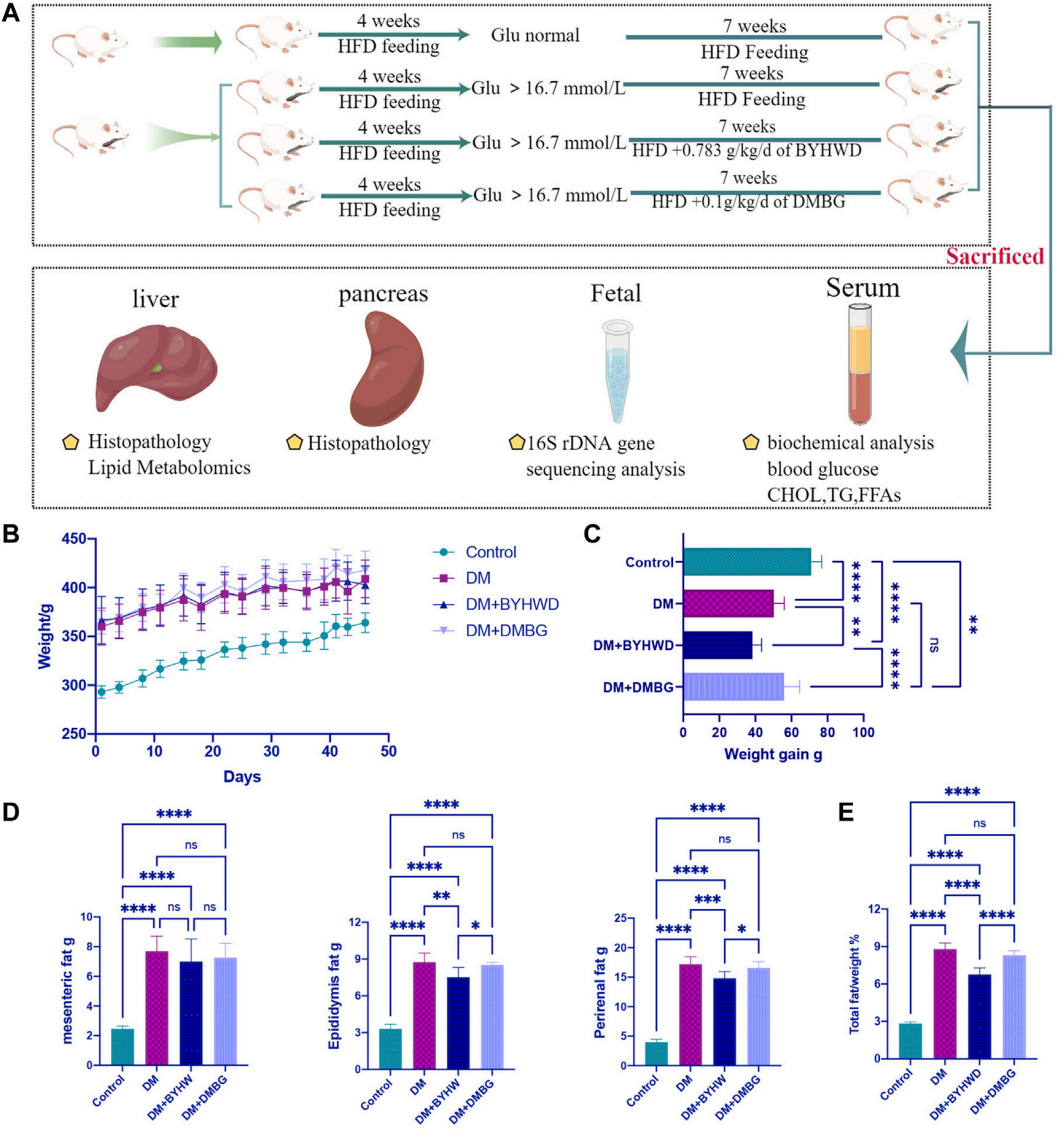
BYHWD attenuated the body weight increase and fat accumulation induced by HFD in diabetic ZDF rats: **(A)** experimental period and treatments’ arrangement; **(B)** average weight change during treatments; **(C)** comparison of weight gain during the experimental period among four different groups; **(D)** comparison on the accumulation of mesenteric fat, epididymis fat, and perirenal fat among four different groups; **(E)** comparison of the ratio of total fat *vs.* body weight among four different groups. Data are shown as means ± S.D (*n* = 6–8). Statistical analysis was performed by using one-way ANOVA, followed by Tukey’s multiple comparisons test for the false discovery rate. *p < 0.05, **p < 0.01, ***p < 0.001, and ****p < 0.0001 were set to compare the mean of each group with the mean of every other group.

The complete model and treatment period lasted 11 weeks (Figure 1A). After 1 week of acclimation with a normal diet, six ZLC rats were fed an HFD as the control group, while 28 ZDF rats were fed an HFD for 4 weeks to establish a diabetic model. Finally, 24 ZDF rats exhibited higher blood glucose levels (>16.7 mmol•L^−1^) and were considered diabetic rats. These 24 diabetic rats were divided into three groups and started on corresponding 7-week drug treatments (DM, BYHWD or DMBG). The ZLC control rats displayed a significant decrease in food and water intake compared to the ZDF rats in the treatment groups suffering T2DM. Each ZLC rat from the control group had only 19.8 ± 1.0 of the average food intakes and 23.3 ± 2.4 of the average water intake. However, ZDF rats with diabetes in the DM group had a significant increase in food and water intake with 40.8 ± 1.9 of average food intake and 112.0 ± 8.1 of average water intake. Both treatments of BYHWD and DMBG could slightly inhibit food and water intake increase induced by the HFD but did not show significant difference when compared to that in the DM group (Supplementary Figures S2A, S2B). The body weight of all rats increased during the experimental period to different extents (Figure 1B). For example, compared with the DM group, BYHWD showed significantly reduced HFD-induced weight gain in ZDF rats (*p* < 0.05, Figure 1C). Conversely, DMBG had no significant effect on HFD-induced weight gain compared with the DM group (Figure 1C). Thus, BYHWD exhibited a better weight reduction effect in ZDF rats than those in the DMBG group (*p* < 0.0001, Figure 1C). In addition, although the ZLC rats gained more weight than the ZDF rats, the average weight of the ZLC rats was considerably lower than that of the ZDF rats in the DM group, with the difference in weight between the two groups being close to 100 g (*p* < 0.0001, Figure 1C). As shown in Figure 1D, the ZDF rats in the three diabetic groups exhibited marked increases in mesenteric fat (*p* < 0.0001), epididymal fat (*p* < 0.0001), and perirenal fat (*p* < 0.0001) compared with the ZLC rats. Among the diabetic ZDF rats, BYHWD treatment did not remarkably decrease mesenteric fat but significantly inhibited HFD-induced epididymal fat (*p* < 0.01, Figure 1D) and perirenal fat accumulation (*p* < 0.001, Figure 1D) in relation to the DM group. Moreover, DMBG did not positively or negatively regulate any of these fats, whereas BYHWD exhibited a better benefit in reducing epididymal fat (*p* < 0.05, Figure 1D) and perirenal fat accumulation (*p* < 0.05, Figure 1D) than DMBG treatment. The percentage of total fat *vs.* body weight reflects the degree of obesity in the rats, and this percentage for the ZDF rats was significantly higher than that for the ZLC rats (*p* < 0.0001, Figure 1E). Although DMBG could decrease the fat-to-body weight ratio, it did not have significant effects compared with the DM group, while BYHWD dramatically decreased the fat-to-body weight ratio compared with the DM (*p* < 0.0001, Figure 1E) or DMBG group (*p* < 0.0001, Figure 1E). All results indicated that BYHWD treatment significantly decreased HFD-induced weight gain and fat accumulation in the ZDF rats and displayed better bioactivity to lose weight and reduce fat than DMBG treatment.

### 3.3 *Buyang Huanwu* decoction decreases the abnormally high blood glucose of high-fat diet-induced T2DM in diabetic ZDF rats

**FIGURE 2:**
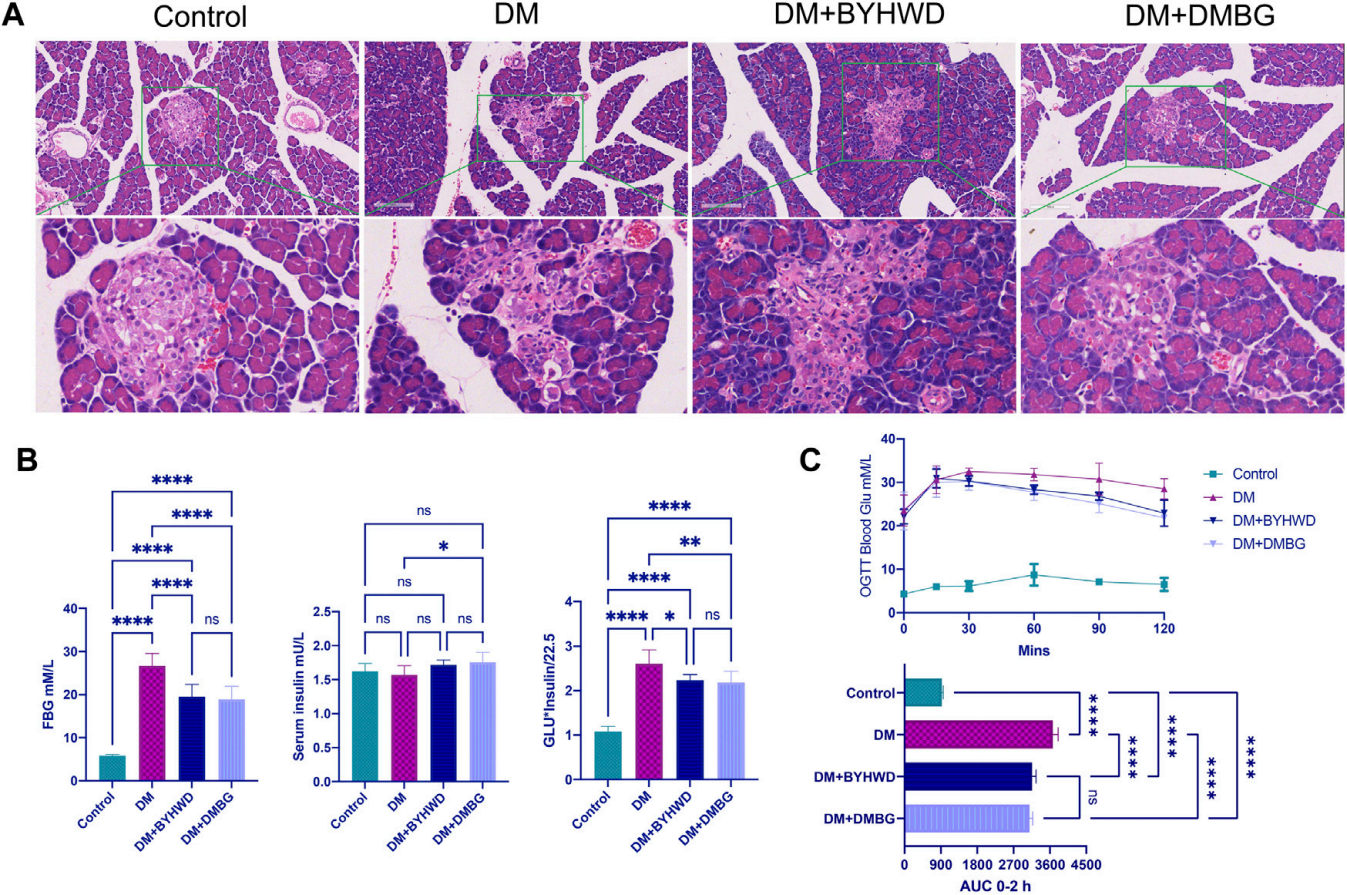
BYHWD maintained the normal structure of the islet and adjusted the abnormal glucose metabolism induced by HFD in ZDF rats: **(A)** representative HE images of the rat pancreas from different groups; **(B)** the comparison of the blood glucose, serum insulin, and the HOMA-IR index among four groups at the end of the treatments; **(C)** the comparison of the results of the OGTT among the four groups during the experimental period. Data are shown as means ± S.D (*n* = 6–8). Statistical analysis was performed using one-way ANOVA, followed by Tukey’s multiple comparisons test for the false discovery rate. *p < 0.05, **p < 0.01, ***p < 0.001, and ****p < 0.0001 were set to compare the mean of each group with the mean of every other group.

Damages to the pancreatic tissue morphology often result in islet dysfunction and T2DM development. Histomorphological examination of the rat pancreas (Figure 2A) revealed that even while feeding on an HFD, the islet structure in the ZLC rats of the control group was relatively normal and the boundary between the islets and exocrine gland was evident on the HE image. Conversely, islets of the diabetic ZDF rats in the DM group were structurally disorganized and irregularly shaped with a notable loss of the normal structure and interlacing growth of internal and external secretory glands. However, after BYHWD treatment, the islets were more regular, and the boundary between the islets and the exocrine gland was clearer than that in the DM group. The DMBG group also exhibited regular islet morphology with a clear boundary between the islets and exocrine glands. Therefore, BYHWD and DMBG treatments were successful in alleviating damage caused to islets and restoring the pathological alterations of the pancreas commonly observed in T2DM rats compared with normal rats.

Furthermore, the ZLC rats in the control group did not exhibit high FBG levels and high AUC of OGTT blood glucose. Conversely, the DM rats exhibited markedly high FBG levels (*p* < 0.0001, Figure 2B), 2.5-fold higher homeostasis model assessment–insulin resistance (HOMA-IR, *p* < 0.0001, Figure 2B), and higher AUC of OGTT blood glucose (*p* < 0.0001, Figure 2C) compared with the ZLC rats in the control group. Fortunately, the BYHWD-treated rats exhibited a marked reduction in FBG levels by 20% of what was observed in the DM rats (*p* < 0.0001, Figure 2B), significantly decreased AUC of OGTT blood glucose (*p* < 0.0001, Figure 2C), and significantly downregulated HOMA-IR (*p* < 0.05, Figure 2B). Relative to the DM rats, DMBG treatment exhibited results similar to BYHWD treatment. In fact, there was no significant difference in the FBG levels, OGTT blood glucose, serum insulin levels, and HOMA-IR between the DMBG and BYHWD groups. This suggested that BYHWD treatment markedly reduced the abnormal glucose with bioactivity similar to the positive control of DMBG treatment.

### 3.4 *Buyang Huanwu* decoction regulates the blood lipid and liver lipid accumulation in diabetic ZDF rats

**FIGURE 3:**
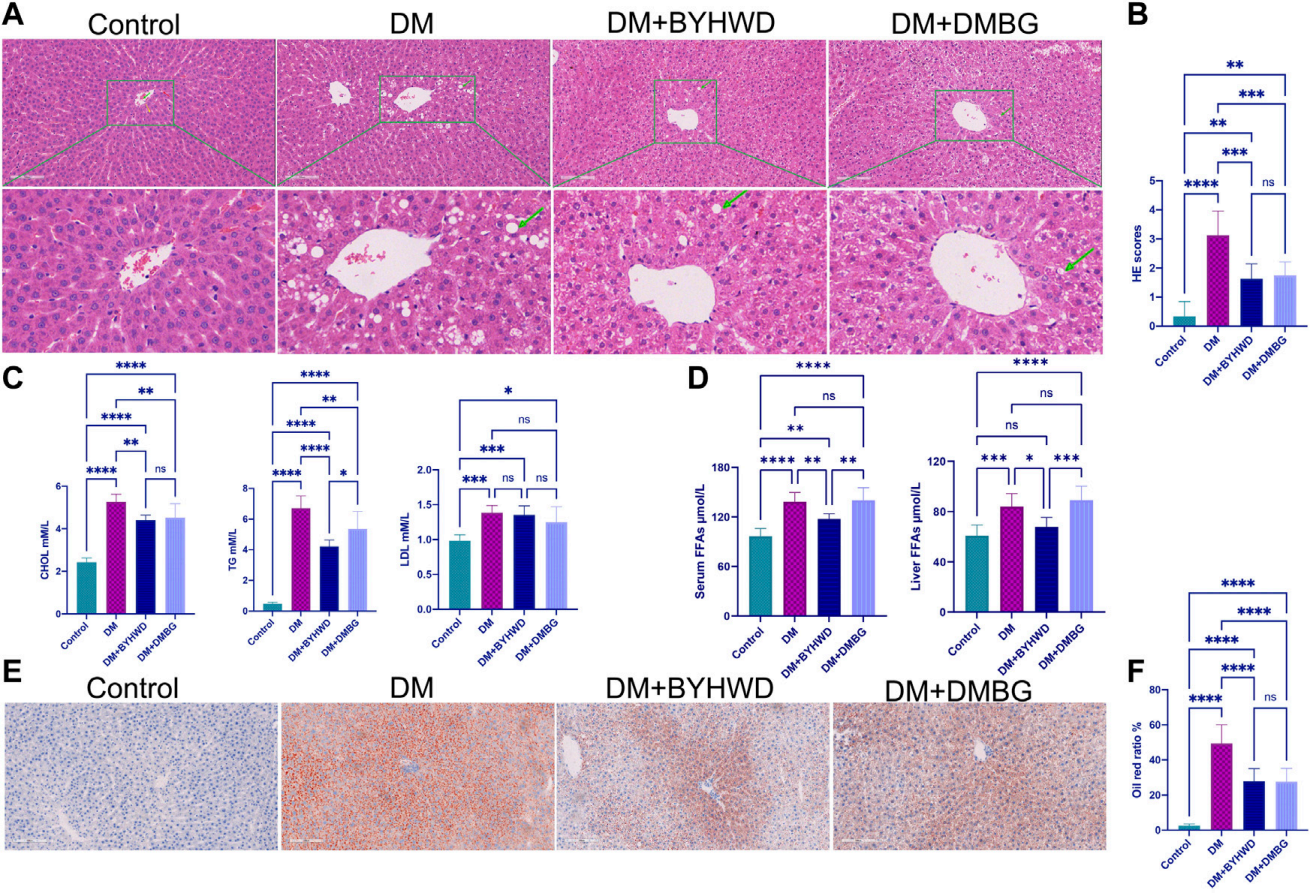
BYHWD decreased the fat accumulation in the liver and reduced the serum lipid levels: **(A)** representative HE images from four groups; **(B)** HE scores’ comparison; **(C)** CHOL, TG, and LDL comparison among the four groups; **(D)** the comparison of FFAs in the liver and in the serum among the four groups; **(E)** representative images of liver tissue section staining by Oil Red O; **(F)** the comparison of the Oil Red O ratio among the four groups. Data are shown as means ± S.D (*n* = 6–8). Statistical analysis was performed by one-way ANOVA, followed by Tukey’s multiple comparisons test for the false discovery rate. *p < 0.05, **p < 0.01, ***p < 0.001, and ****p < 0.0001 were set to compare the mean of each group with the mean of every other group.

Fatty liver and hyperlipidemia are the very important clinicopathological bases of obesity and T2DM. HE staining of the rat liver tissue revealed (Figure 3A) that even after feeding on the same HFD, the liver of the ZLC rats depicted a complete structure, with clear liver cells and a well-arranged cord structure. In addition, the shape and size of the cells were normal without notable lesions. However, the diabetic ZDF rats from the DM group displayed an incomplete array of the hepatic cord structure, loosening of the cytoplasm of liver cells, steatosis of numerous liver cells with a wide range, and different sizes of fat droplets. Fortunately, BYHWD markedly decreased HFD-induced abnormal pathological alterations in the liver tissue. With BYHWD treatment, the liver lobules showed relatively intact structures, with neatly arranged cords, clear cell nuclei, and slight steatosis of liver cells with remarkably fewer fat droplets. Alterations in the liver morphology with DMBG treatment were similar to those with BYHWD treatment, except for some liver cells that exhibited mild steatosis. As shown in Figure 3B, both treatments dramatically reduced the HE scores (*p* < 0.001), but no significant difference was observed between both treatments.

Moreover, the ZDF rats exhibited high blood glucose levels and hyperlipidemia after feeding on the HFD because the levels of the key serum lipid markers changed dramatically. For example, the CHOL, TG, and LDL levels in the DM group were 2.2-fold (*p* < 0.0001, Figure 3C), 14-fold (*p* < 0.0001, Figure 3C), and 1.4-fold (*p* < 0.001, Figure 3C) higher than those in the control group, respectively. Compared with the DM group, BYHWD treatment improved the hyperlipidemic state through a significant reduction in serum lipid marker levels as follows: approximately one-third of TG (*p* < 0.0001, Figure 3C) and one-fifth of CHOL (*p* < 0.01, Figure 3C). DMBG also significantly decreased the TG (*p* < 0.001, Figure 3C) and CHOL (*p* < 0.05, Figure 3C) levels, but BYHWD treatment displayed a significantly better benefit in TG reduction than DMBG treatment (*p* < 0.05, Figure 3C). Unfortunately, both treatment groups exhibited a slight decrease in LDL compared with the DM group; the difference was non-significant.

Meanwhile, free fatty acids (FFAs) were the other crucial factor that resulted in hyperlipidemia. As shown in Figure 3D, the BYHWD-treated group exhibited a significant reduction in the HFD-induced increase in serum FFAs (*p* < 0.01) and liver FFAs (*p* < 0.05) compared with the DM group. The DMBG-treated group also exhibited a decrease in both FFAs compared with the DM group; however, the decrease was non-significant. BYHWD treatment decreased more serum FFAs (*p* < 0.01) and liver FFAs (*p* < 0.001) than DMBG treatment. The observed data were also verified through Oil Red O staining for different groups (Figure 3E). The representative images revealed that the liver of DM rats was fully packed with Oil Red O-stained lipid droplets. However, with BYHWD and DMBG treatments, the number of lipid droplets was reduced and replaced with DAPI-stained normal cell nuclei. Meanwhile, through semi-quantitative lipid drop analysis (proportion of the red part area/total area), we found that the lipid droplets in the DM rats were 20-folds higher than those in the control rats (*p* < 0.0001, Figure 3F). However, with BYHWD or DMBG treatment, the number of lipid droplets in the diabetic rats was reduced to 60% of the original value (*p* < 0.01, Figure 3F). No significant difference was observed between both BYHWD and DMBG groups.

### 3.5 *Buyang Huanwu* decoction meliorates high-fat diet-induced gut microbiota dysbiosis in diabetic ZDF rats

**FIGURE 4:**
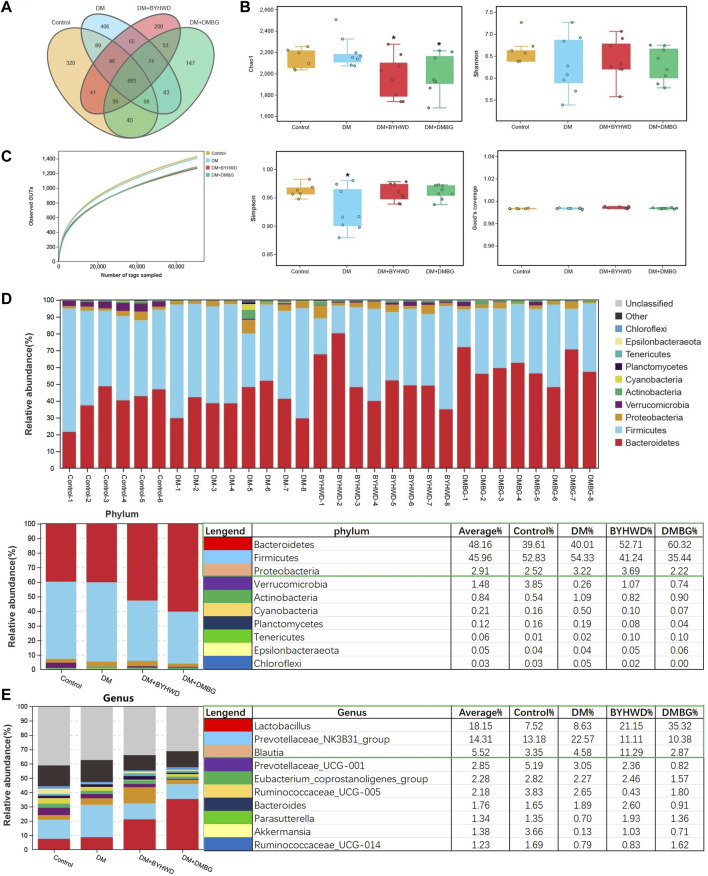
Influences of BYHWD on gut microbiota in diabetic ZDF rats: **(A)** Venn analysis; **(B)** alpha-indexes of Chao1, Shannon, Simpson, and Good’s coverage comparison among the four groups; **(C)** observed OTUs; **(D)** the histogram of the top 10 phyla of the relative abundance of the intestinal flora; **(E)** the histogram of the top 10 genera of the relative abundance of the intestinal flora. Data are shown as means ± S.D (*n* = 6–8). Statistical analysis was performed using one-way ANOVA, followed by Tukey’s multiple comparisons test for the false discovery rate. *p < 0.05 and **p < 0.01 were set to compare the mean of each group with the mean of the DM group.

The obtained DNA fragments were sequenced using MisSeq PE300 based on 16S rDNA sequencing results. The original sequences that passed the initial quality screening were divided into libraries and samples. The barcode sequences were removed to generate effective tags for cluster OTUs. The effective tags from each group were 97,9233 ± 2,129 of the control group, 99,277 ± 5,880 of the DM group, 98,473 ± 2,603 of the BYHWD group, and 93,708 ± 3,398 of the DMBG group. These effective tags were clustered using Uparse software for OTUs on the basis of 97% similarity. The abundance of OTU preliminarily indicates the species richness of the bacterial community in the sample. Thus, Venn diagram analysis was conducted to fully understand overlapping and different OTUs among the groups. According to the Venn analyses shown in Figure 4A, four groups shared 693 OTUs. Moreover, the control fecal samples contained 320 unique OTUs, while the other three groups comprised 406 unique OTUs of the DM group, 200 unique OTUs of the BYHWD group, and 147 unique OTUs of the DMBG group.

The alpha-diversity indices including Chao1, Shannon, and Simpson were used to evaluate the richness and diversity of the gut flora. As shown in Figure 4B, among the four groups, the Chao1 index of the control group was 2,139.49 ± 94.75 and that of the DM group slightly increased (2202.87 ± 144.52) without any significant difference. However, compared with the DM group, the Chao1 index in the BYHWD and DMBG groups was slightly decreased (1971.475 ± 202.44 (*p* < 0.05, Figure 4B) and 1986.76 ± 186.37 (*p <* 0.05, Figure 4B), respectively). The control group had the largest Shannon index (6.66 ± 0.33), whereas the DM group had the smallest Shannon index (6.30 ± 0.65). The BYHWD and DMBG groups (6.41 ± 0.48 and 6.31 ± 0.39, respectively) had slightly higher Shannon indices than the DM group. However, no significant difference in the Shannon index was observed among the four groups. The control, BYHWD, and DMBG groups had similar Simpson indices of 0.96 ± 0.012, 0.96 ± 0.013, and 0.96 ± 0.016, respectively, while the Simpson index of the DM group was 0.93 ± 0.038, slightly lower than those in the other groups (*p* < 0.05, Figure 4B). This suggested the control, BYHWD, and DMBG groups had a higher diversity of intestinal flora. The Good’s coverage indices were nearly >98% among the four groups, demonstrating that most of the gut microbial diversities have been captured in the present study. The observed OTUs were almost equal and revealed the sequencing effort that was sufficient to describe the gut microbiota diversity of the samples (Figure 4C). The top 10 phyla in the samples were *Bacteroidetes*, *Firmicutes*, *Proteobacteria*, *Verrucomicrobia*, *Actinobacteria*, *Cyanobacteria*, *Planctomycetes*, *Tenericutes*, *Epsilonbacteraeota*, and *Chloroflexi*, in that order (Figure 4D). Among these top 10 phyla, *Bacteroidetes*, *Firmicutes*, and *Proteobacteria* were the dominant phyla with relative abundances of 48.16%, 45.96%, and 2.91%, respectively (Figure 4D). Compared with the DM group, the relative abundance of *Bacteroidetes* significantly increased, whereas that of *Firmicutes* dramatically decreased in the BYHWD- and DMBG-treated rats. Meanwhile, *Proteobacteria* had a higher relative abundance in the BYHWD group (Figure 4D). In addition, the top 10 genera in the samples in the order were *Lactobacillus*, *Prevotellaceae_NK3B31_group*, *Blautia*, *Prevotellaceae_UCG-001*, *Eubacterium_coprostanoligenes_group*, *Ruminococcaceae_UCG-005*, *Bacteroides*, *Parasutterella*, *Akkermansia*, and *Ruminococcaceae_UCG-014* (Figure 4E). Among these top 10 genera, *Lactobacillus*, *Prevotellaceae_NK3B31_group*, and *Blautia* were the preponderate genera with relative abundances of 18.15%, 14.31%, and 5.52%, respectively. Compared with the DM group, BYHWD significantly increased the relative abundance of *Lactobacillus* from 8.63% of DM to 21.15% and increased the relative abundance of *Blautia* from 4.58% of DM to 11.29%. Moreover, BYHWD significantly decreased the relative abundance of *Prevotellaceae_NK3B31_group* from 22.57% of DM to 11.11%. DMBG had the same effect as BYHWD in increasing the relative abundance of *Lactobacillus* to 35.32% and significantly decreasing the relative abundance of the *Prevotellaceae_NK3B31_group* to 10.38%. However, its effect was opposite to that of BYHWD when it decreased the relative abundance of *Blautia* from 4.58% of DM to 2.87%.

**FIGURE 5:**
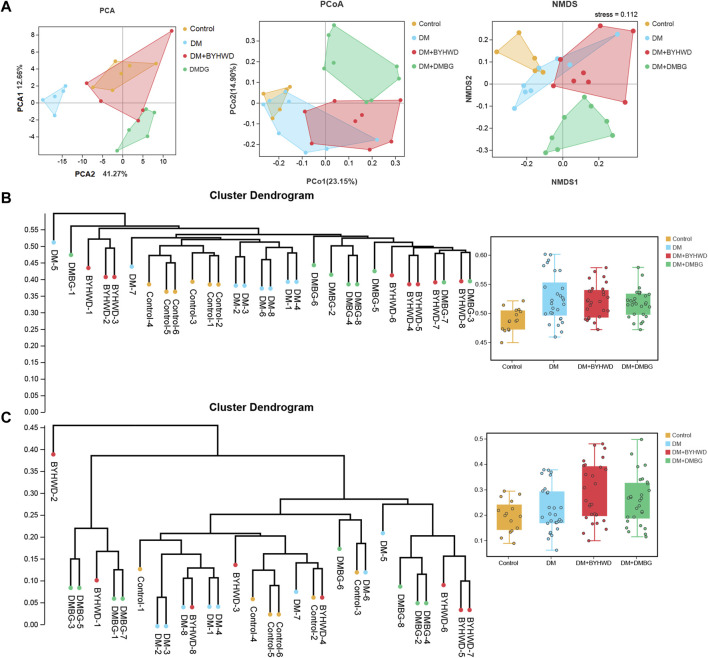
Spearman’s correlations among the pharmaceutical factors, the differential lipid metabolites, and the gut microbiome in diabetic ZDF rats after BYHWD treatment: **(A)** associations between the pharmaceutical factors and the differential lipid metabolites; **(B)** upper panel exhibits the associations between the pharmacological factors and the gut microbiome at the phylum level, while the lower panel displays the associations between the differential lipid metabolites and the gut microbiome at the phylum level; **(C)** the upper panel exhibits the associations between the pharmacological factors and the gut microbiome at the genus level, while the lower panel displays the associations between differential lipid metabolites and the gut microbiome at the genus level. The magnitude of the correlation is represented by the color intensity (blue, negative correlation; red, positive correlation), and the asterisks (*) in correlation heatmaps indicate *p* < 0.05.

**FIGURE 6:**
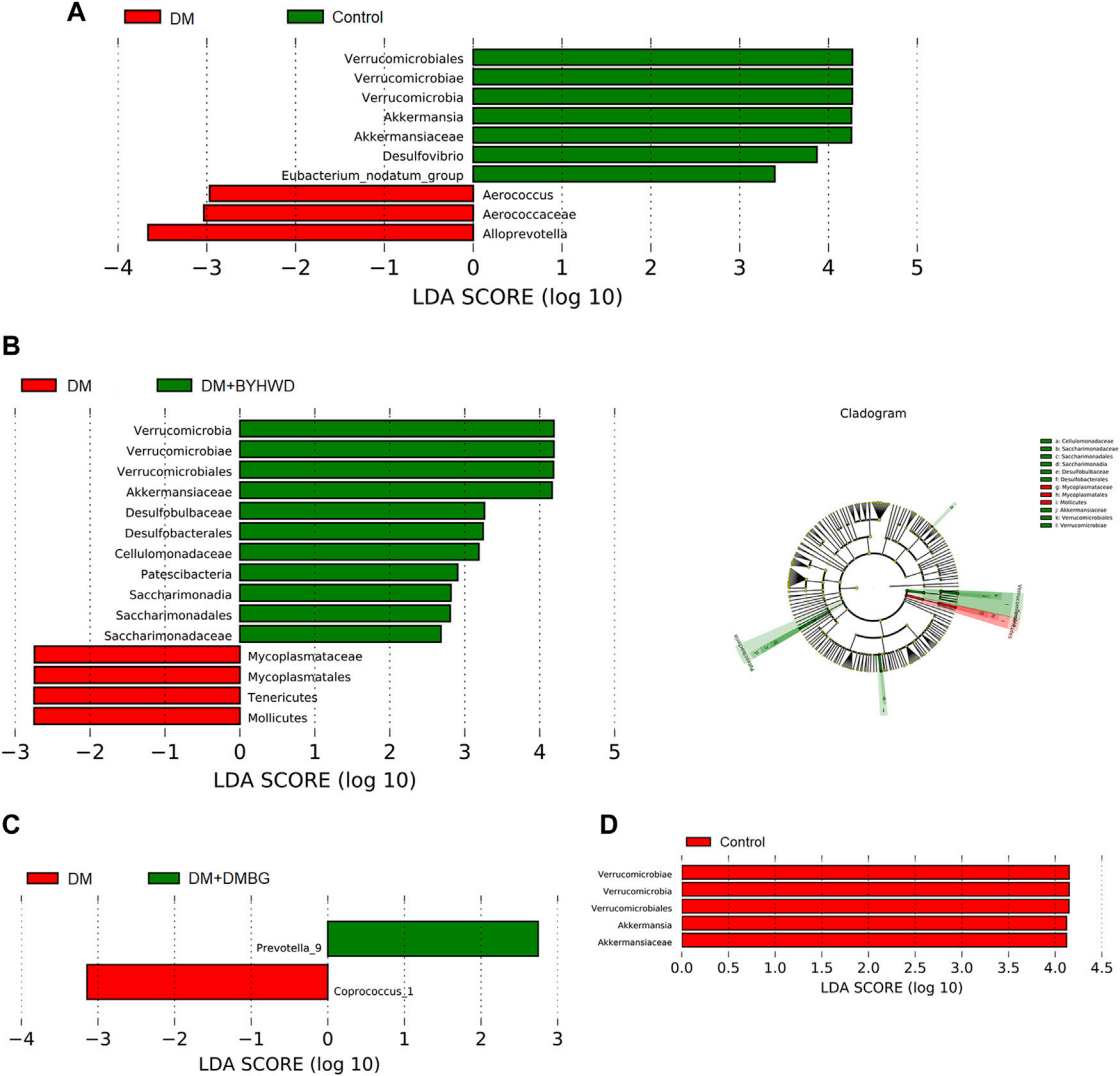
LEfSe analysis of the gut flora composition of the four study groups (LEfSe analysis exhibited differentially abundant taxa with *p* < 0.05 and LDA score >2.0.); **(A)** LEfSe analysis results between the DM and control groups; **(B)** LEfSe analysis results between the DM and BYHWD groups; **(C)** LEfSe analysis results between the DM and DMBG groups; **(D)** LEfSe analysis results between the BYHWD and control groups.

**FIGURE 7:**
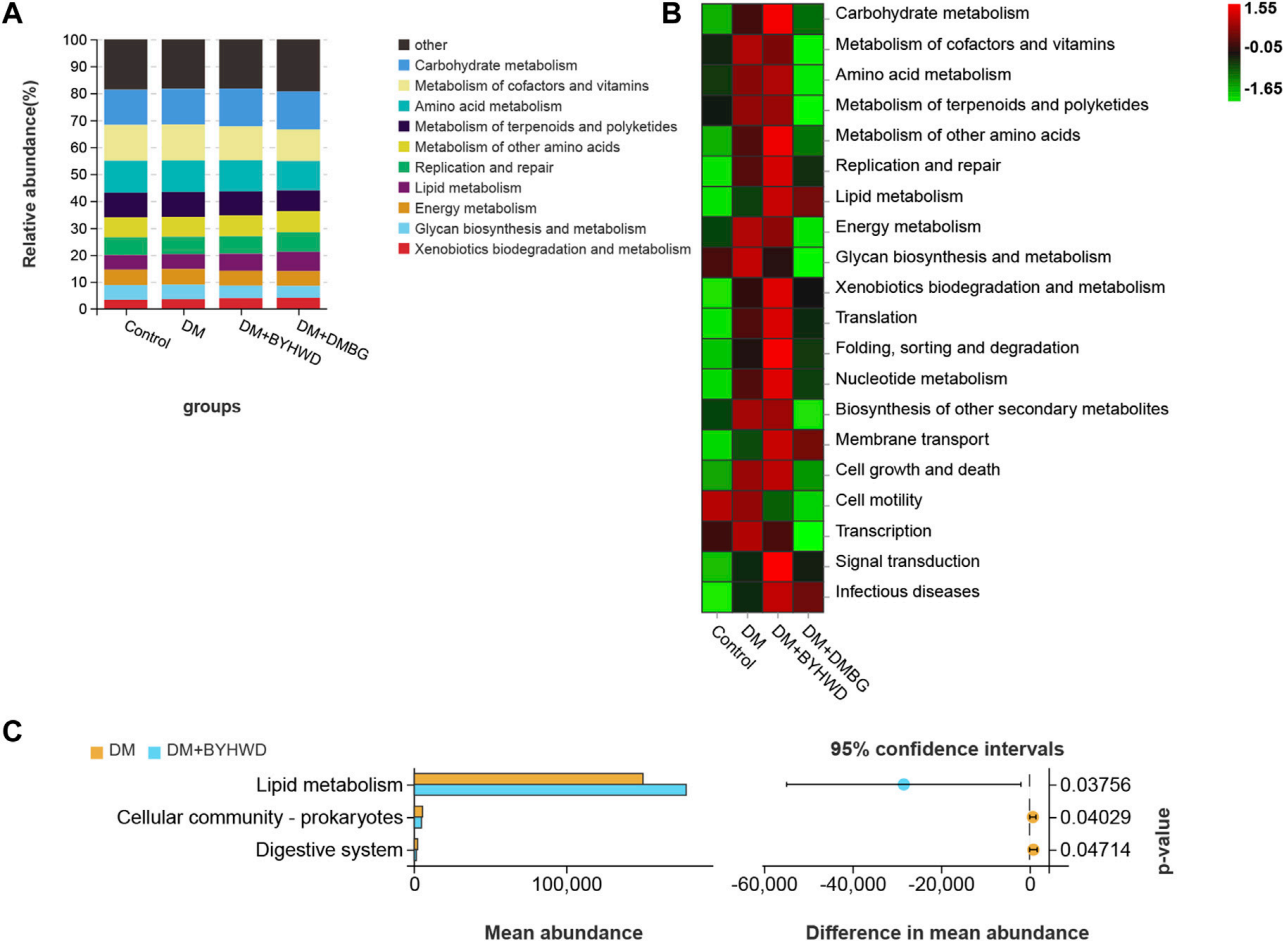
© The effect of BYHWD treatment on the composition and diversity of the gut flora: **(A)** bacterial beta-diversity among the four groups was accessed by PCA, PCoA, and NMDS; **(B)** the cluster dendrogram of all samples based on the weight UniFrac distance and comparison of each group; **(C)** the cluster dendrogram of all samples based on the unweight UniFrac distance and comparison of each group. Data are shown as means ± S.D (*n* = 6–8). Statistical analysis was performed by using one-way ANOVA, followed by Tukey’s multiple comparisons test for the false discovery rate. *p < 0.05 was set to compare the mean of each group with the mean of the DM group.

Next, beta-diversity analysis was performed to further investigate the effect of BYHWD on the variations in the gut flora community. PCA, PCoA, and NMDS analyses revealed that the plots of the samples from the BYHWD group were clustered together in the red region and apart from the region of the DM and DMBG groups (Figure 5A). Particularly, both regions of the BYHWD and control groups nearly overlapped in the PCA, indicating that BYHWD changed the structure and composition of HFD-induced abnormal intestinal flora in the diabetic ZDF rats. Moreover, based on the unweighted and weighted UniFrac distances, UPGMA indicated that four different groups had a certain separation of the microbiota from each other. The BYHWD and DMBG groups showed a significant difference in both distances with the DM group (*p* < 0.05, Figures 5B,C). At the same time, the LeFSe analysis was used to identify primary changes in intestinal flora taxa. The results in Figure 6 showed a difference in the relative abundance of the major different gut bacterial taxa compared to each group with every other group. On one hand, the results indicated that diabetes significantly changed the intestinal flora and resulted in gut dysbiosis. For example, the genera of *Akkermansiae*, *Desulfovibrio*, and *Eubacterium_nodatum_group* were enriched in the fecal samples from the control group, and *Aerococcus* and *Alloprevotella* were enriched in the fecal samples from the DM group (Figure 6A). On the other hand, BYHWD could meliorate HFD-induced gut dysbiosis. For instance, the phyla of *Verrucomicrobia*, *Patescibacteria*, and *Saccharimonadia* and the families of Akkermansiaceae, Desulfobulbaceae, and Callulomonadaceae were more abundant in the fecal samples from the BYHWD group compared with the DM group (Figure 6B). Conversely, few indicators revealed that different taxa existed between the DM and DMBG groups, with only the genus *prevotella_9* from the DMBG group exhibiting abundance compared with the DM group (Figure 6C). No different taxa were displayed by the BYHWD group compared with the control group (Figure 6D). Moreover, these indicative different gut flora in response to the relative abundance of different functional categories were further investigated using the KEGG database. These relatively abundant different functional categories in different groups were majorly involved in processes such as carbohydrate metabolism, metabolism of cofactors and vitamins, amino acid metabolism, metabolism of terpenoids and polyketides, metabolism of other amino acids, replication and repair, lipid metabolism, and energy metabolism (Figures 7A,B). Notably, the dominant different metabolism between the DM and BYHWD groups was lipid metabolism (Figure 7C); this result is consistent with those described in Section 3.2 and Section 3.3. This also strongly indicated the effect of BYHWD in reducing fat accumulation, improving abnormal lipid metabolism, and meliorating the T2DM-induced abnormal abundance of the intestinal microbiota.

### 3.6 *Buyang Huanwu* decoction leads to alterations in liver lipid metabolites in diabetic ZDF rats

**FIGURE 8:**
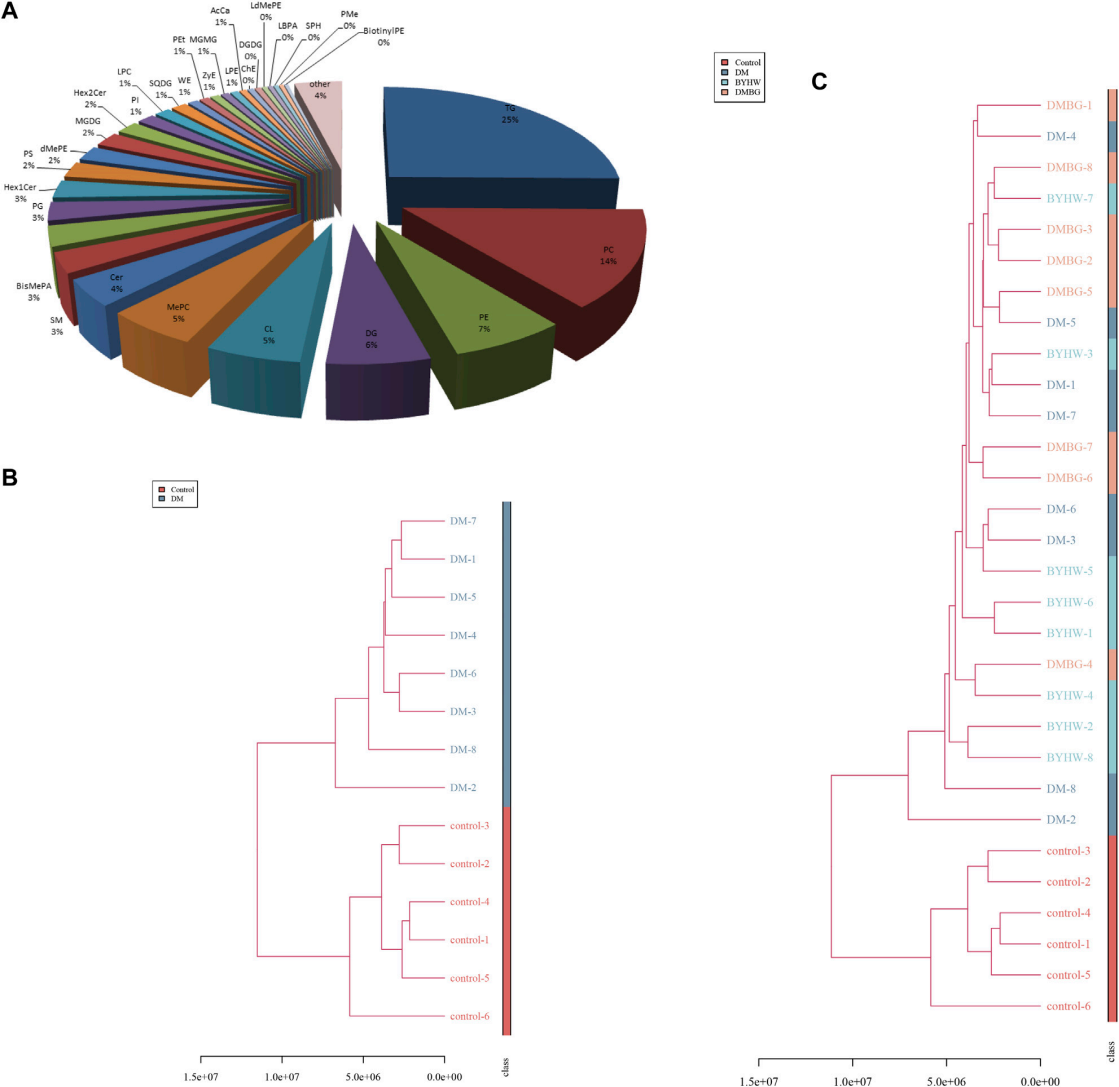
KEGG analysis for BYHWD involved indicative different gut florae in response to the relative abundance of different functional categories: **(A)** the histogram of the related functional categories of the four groups; **(B)** the heatmap of related functional categories of the four groups; **(C)** significantly different functional categories between the DM and BYHWD groups.

The liver is a central organ that maintains blood glucose stability and normal lipid metabolism. It is also a major target organ of insulin action. Abnormal hepatic lipid metabolism is now considered a manifestation of T2DM and metabolic syndrome (Li et al., 2016). In diabetic patients with abnormal lipid metabolism in the liver, insulin sensitivity is reduced, liver glucose metabolism is impaired, hepatoproglucose production is increased, and hepatic glucose production is increased, which together lead to a significant increase in the blood glucose levels, especially FBG, which eventually results in accelerated fat accumulation in the liver (Roden and Bernroider, 2003). Therefore, based on the aforementioned prediction, we further conducted a liver lipid metabolomics study to explore the BYHWD-induced changes in specific lipid metabolites in diabetic ZDF rats. Based on our liquid chromatography–mass spectrometry analysis, 3,498 lipids were annotated and normalized into the matrix for further analysis. As shown in Figure 8A, among these 3,498 identified lipids, the number of TGs was the highest, and TGs accounted for 25% of the total lipids. The top 10 detected lipids were TGs (TC, 25%), phosphatidylcholines (14%), phosphatidylethanolamines (7%), diglyceride (6%), cardiolipins (5%), methyl phosphatidylcholine (5%), ceramides (4%), sphingomyelin (3%), bis-methyl phosphatidic acids (BisMePA, 3%), and phosphatidylglycerols (3%), in that order. Next, the liver samples from different treatment groups were analyzed to assess similarities and form a hierarchical cluster. Figure 8B shows that six samples from the control group had shorter Euclidean distances and were clustered together. In the meantime, eight samples from the DM group clustered into a family. These results revealed that the lipids in these two groups were distinct and could potentially be used to differentiate the two groups. Calculation of the Euclidean distance of all samples from the four groups revealed that the six samples from the control group initially formed a cluster at the bottom of the dendrogram image, whereas the remaining 24 samples (eight samples from each group) from rats with T2DM from the DM, BYHWD, and DMBG groups formed another cluster, shown in Figure 8C. In this larger cluster, six samples from the BYHWD group showed the same short Euclidean distance and were clustered together with five samples from the DMBG group. The DM samples, however, were found scattered with the BYHWD and DMBG samples. These findings suggest that both drug groups had common features and shared unique characteristics of lipid metabolism.

**FIGURE 9:**
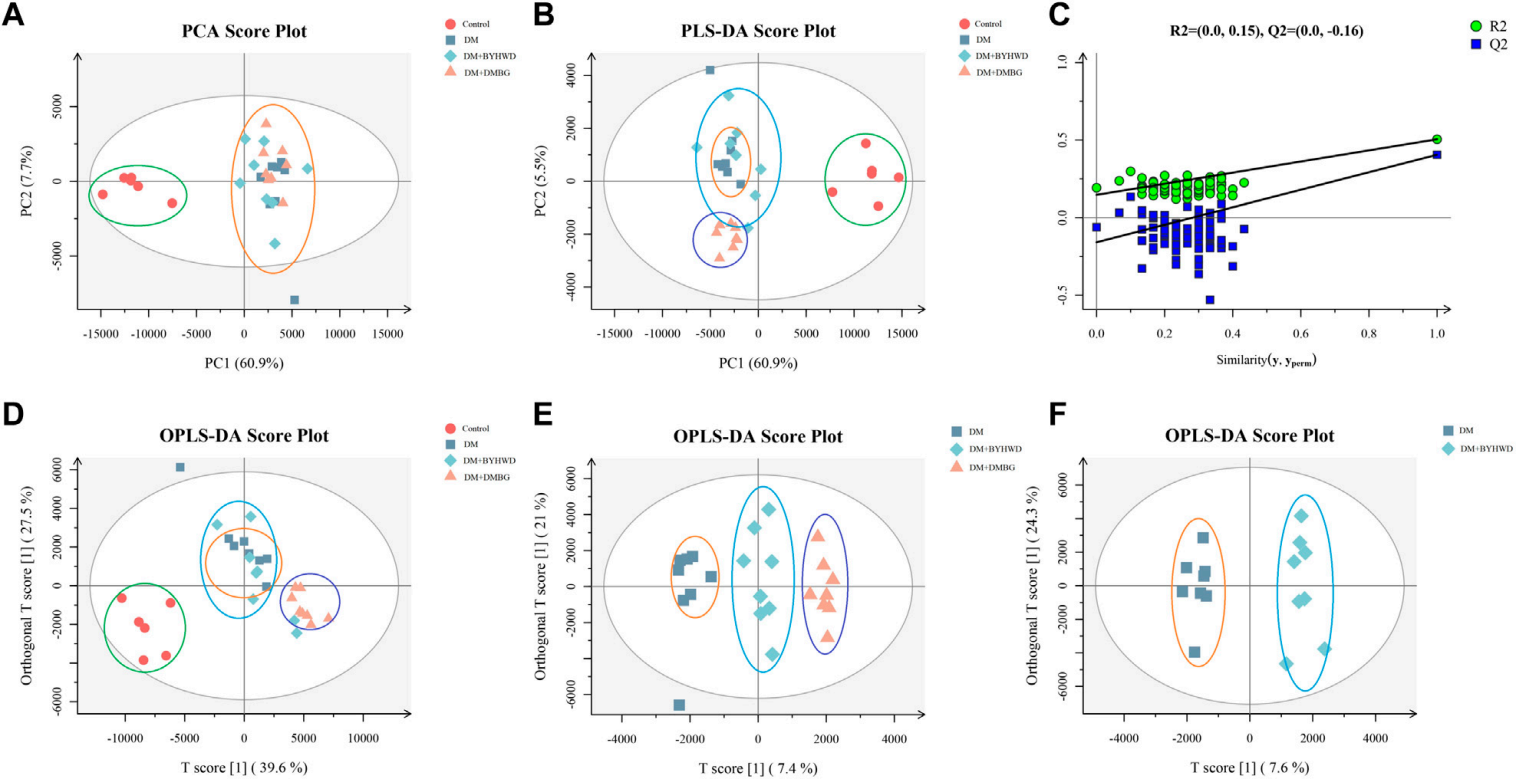
BYHWD affected the lipid metabolism displayed by multivariate statistical analysis: **(A)** PCA plots of the four study groups; **(B)** PLS-DA plots of the four groups; **(C)** permutations plots of the four study groups; **(D)** OPLS-DA plots of the four study groups; **(E)** OPLS-DA plots of DM, DM + BYHWD, and DM + DMBG groups; **(F)** OPLS-DA plots of the DM and DM + BYHWD groups. The red circle represents the rat’s sample from the control group; the deep-blue square represents the rats’ samples from the DM group; the light-blue diamond represents the rats’ samples from the DM + BYHWD group, and the orange triangle represents the rats’ samples from the DM + DMBG group.

**FIGURE 10:**
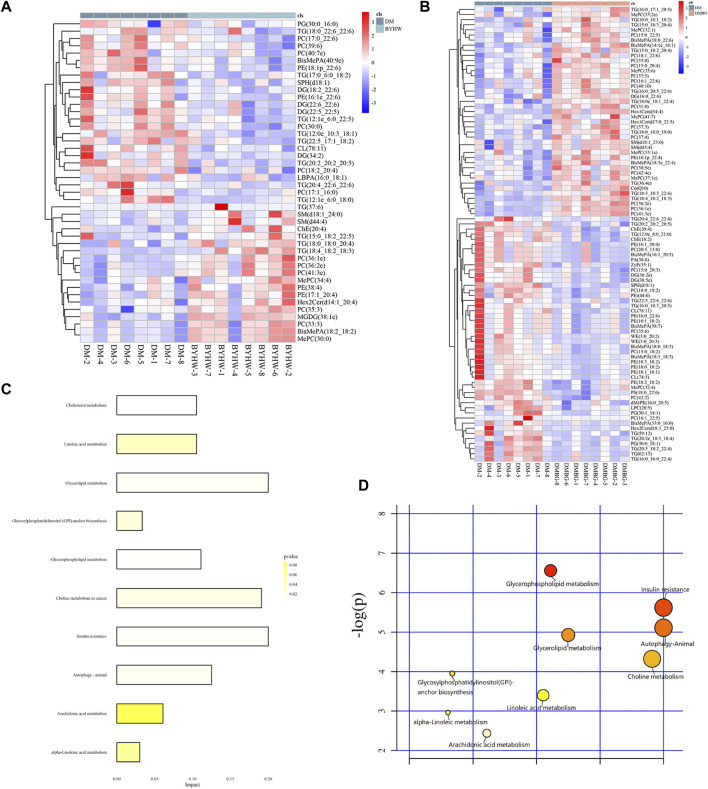
BYHWD affected the lipid classification identified by the hierarchical cluster analysis: **(A)** lipid classification identified by LC-Orbitrap MS; **(B)** hierarchical cluster analysis between the control and DM groups; control-1 to control-6 represent the liver samples of six rats from the control group, and DM-1 to DM-8 represent the liver samples of eight rats from the DM group; **(C)** hierarchical cluster analysis among the four study groups. BYHW-1 to BYHW-8 represent the liver samples of eight rats from the DM + BYHWD group, and DMBG-1 to DMBG-8 represent the liver samples of eight rats from the DM + DMBG group.

To further explore metabolic distinctions among the four groups, the principal component analysis (PCA), partial least squares-discriminant analysis (PLS-DA), and orthogonal least squares-discriminant analysis (OPLS-DA) were performed. As shown in Figure 9A, the PCA plots demonstrated that all samples were divided into two different zones. The plots of the control group were on the left X-axis and far away from those of the three groups with T2DM. Hence, the ZLC rats were demonstrated to have a completely different pattern of lipid metabolism compared with the ZDF rats with diabetes. However, the plots of the ZDF rats from the two treated groups were clustered together with those of the DM group, without any notable distinction between the treated and untreated groups in the PCA. Therefore, the PLS-DA was conducted for the distinct three groups ulteriorly (Figure 9B). The PLS-DA plots of the control groups were also distinctly separate from those of the three groups with T2DM. The plots of the three groups with T2DM did not cluster like those in the PCA. Moreover, the plots of the DMBG group gathered together in a dependent zone. In addition, the plots of the DM and BYHWD groups showed relatively better distribution from one another, compared with those in the PCA. In Figure 9C, the permutations plot simultaneously indicates that the PLS-DA model is suitable for differentiating the four groups and do not overfit. To further differentiate the plots of the DM and BYHWD groups, and screen for potential pharmacological biomarkers, OPLS-DA was used as an extension of PLS-DA. In Figure 9D, the plots of the control group have a very clear separation from the remaining plots. Additionally, the three groups with T2DM were completely separated from each other and the plots of the same group were clustered together (Figure 9E). In particular, the BYHWD plots were always closer to the DM plots in the PCA and PLS-DA but were well separated in the OPLS-DA (Figure 9F). Moreover, 44 discriminately altered lipid metabolites were observed between the DM and BYHWD groups. Among these 44 metabolites, the BYHWD group showed significantly decreased levels of 25 lipid metabolites and remarkably increased levels of 19 lipid metabolites compared with the DM group (Table 1). Furthermore, hierarchical clustering was performed to visualize the relationship and differences in the relative levels of differential metabolites among the three groups with T2DM. Heatmaps in Figures 10A,B suggest that HFD-induced lipid metabolic profiles in the ZDF rats alternate after 7 weeks of BYHWD and DMBG treatments. The metabolism pathways of these differential lipids between the DM and BYHWD groups were further explored using the functional enrichment analysis. The KEGG pathway revealed 10 major metabolic pathways related to the difference between the DM and BYHWD groups. These metabolic pathways, namely, cholesterol metabolism, linoleic acid metabolism, glycerolipid metabolism, glycosylphosphatidylinositol anchor biosynthesis, glycerophospholipid metabolism, choline metabolism in cancer, insulin resistance, autophagy-animal, arachidonic acid metabolism, and alpha-linoleic acid metabolism, may be linked to the role of BYHWD in regulating glycose and lipid metabolism (Figures 10C,D). Overall, these findings implied that the lipid metabolic profile perturbed by T2DM could be meliorated by BYHWD treatment.

**TABLE 1:** Significantly different qualitative lipid metabolites in the liver samples from ZDF rats after BYHWD treatment.

Lipid metabolite	VIP^a^	Fold-change^b^	log2(FC_DM/BYHW)^c^	*p*-value^d^
Increased				
TG (37:6)	1.22	0.21	−2.24	0.031
TG (18:4_18:2_18:3)	2.41	0.26	−1.94	0.003
PC (36:1e)	1.12	0.59	−0.77	0.005
PC (36:2e)	2.82	0.64	−0.64	0.018
PE (38:4)	1.12	0.64	−0.64	0.024
PE (17:1_20:4)	1.37	0.73	−0.46	0.041
PC (41:3e)	3.33	0.73	−0.45	0.018
TG (18:0_18:0_20:4)	1.08	0.74	−0.43	0.024
ChE (20:4)	1.58	0.75	−0.42	0.031
SM (d18:1_24:0)	2.29	0.76	−0.39	0.018
PC (35:3)	5.01	0.77	−0.37	0.018
MGDG (38:1e)	1.23	0.80	−0.31	0.004
BisMePA (18:2_18:2)	4.74	0.82	−0.29	0.014
MePC (30:0)	4.74	0.82	−0.29	0.014
SM (d44:4)	1.69	0.84	−0.25	0.031
PC (33:3)	4.18	0.85	−0.23	0.014
Hex2Cer (d14:1_20:4)	1.14	0.86	−0.22	0.041
TG (15:0_18:2_22:5)	1.30	0.88	−0.18	0.041
MePC (34:4)	7.54	0.94	−0.09	0.041
Decreased				
TG (20:4_22:6_22:6)	2.08	2.16	1.11	0.031
TG (12:1e_6:0_18:0)	1.30	1.95	0.97	0.024
DG (34:2)	1.70	1.85	0.89	0.041
PC (17:1_16:0)	1.30	1.85	0.89	0.018
TG (17:0_6:0_18:2)	1.20	1.81	0.86	0.041
TG (20:2_20:2_20:5)	1.51	1.53	0.61	0.004
DG (22:5_22:5)	1.03	1.51	0.60	0.031
TG (12:1e_6:0_22:5)	1.20	1.52	0.60	0.014
DG (18:2_22:6)	2.86	1.47	0.56	0.024
DG (22:6_22:6)	4.64	1.39	0.48	0.005
TG (22:5_17:1_18:2)	1.45	1.29	0.37	0.041
CL (78:11)	3.50	1.27	0.35	0.014
PC (30:0)	1.98	1.26	0.33	0.024
SPH (d18:1)	1.19	1.22	0.29	0.031
LBPA (16:0_18:1)	1.78	1.18	0.24	0.041
PC (40:7e)	1.08	1.18	0.24	0.041
PC (17:0_22:6)	1.02	1.17	0.22	0.007
PC (18:2_20:4)	2.03	1.15	0.21	0.018
PC (39:6)	3.86	1.16	0.21	0.041
PE (16:1e_22:6)	1.10	1.16	0.21	0.024
BisMePA (40:9e)	1.20	1.13	0.18	0.014
TG (18:0_22:6_22:6)	1.32	1.13	0.18	0.041
PE (18:1p_22:6)	1.13	1.12	0.16	0.014
PG (30:0_16:0)	4.75	1.11	0.16	0.031

^a,d^ Significantly different lipid metabolites with VIP values > 1.0 and *p* < 0.05 were selected. ^b,c^Fc indicates the fold-change calculated as the relative altered ratio of significantly different lipid metabolites between the BYHWD and DM groups.

### 3.7 Possible correlation among the pharmacological factors, the gut flora, and lipid metabolites in diabetic ZDF rats undergoing *Buyang Huanwu* decoction treatment

**FIGURE 11:**
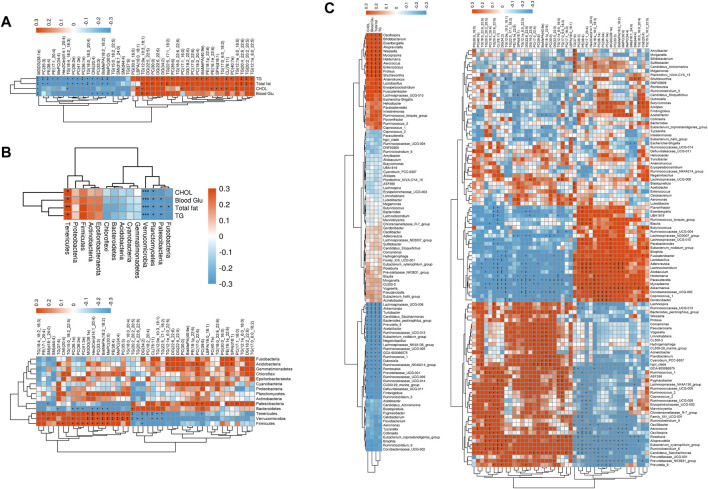
BYHWD improved the metabolic pattern and the level of the differential lipids induced by the T2DM model: **(A)** the heatmap of the differential lipids between the DM and DM + BYHWD groups; **(B)** the heatmap of the differential lipids between the DM and DM + DMBG groups; **(C)** KEGG enrichment pathways of significant differential lipids in the DM + BYHWD group when compared with that in the DM group; **(D)** significant metabolic pathways map in the DM + BYHWD group when compared with that in the DM group.

First, four key pathological indicators (i.e., blood glucose, total fat, CHOL, and TG) closely associated with T2DM were selected as the major pharmacological factors, and Spearman’s correlational analysis was applied to examine the relationships between the pharmacological factors and the lipid metabolites. As shown in Figure 11A, a total of 13 lipids [TG (12:1e_6:0_22:5), TG (20:2_20:2_20:5), TG (20:4_22:6_22:6), TG (12:1e_6:0_18:0), PC (40:7e), CL (78:11), SPH (d18:1), PE (18:1p_22:6), BisMePA (40:9e), PC (18:2_20:4), DG (34:2), DG (22:6_22:6), and PC (30:0)] were positively and 13 lipids [MGDG (38:1e), PC (35:3), PE (38:4), PE (17:1_20:4), MePC (34:4), TG (18:4_18:2_18:3), PC (41:3e), PC (36:2e), PC (36:1e), TG (18:0_18:0_20:4), ChE (20:4), PC (33:3), BisMePA (18:2_18:2), and MePC (30:0)] were negatively associated with TG. Except PE (17:1_20:4), MePC (34:4), and PC (33:3), 11 of the 13 negative lipids to TG were found to be negatively associated with the total fat, while 10 lipids, which included six same lipids to TG [(TG (12:1e_6:0_22:5), TG (20:2_20:2_20:5), TG (20:4_22:6_22:6), TG (12:1e_6:0_18:0), DG (34:2), PC (30:0) and four unique lipids of TG (12:0e_10:3_18:1), DG (22:5_22:5), DG (18:2_22:6), TG (12:1e_6:0_18:0)] that were positively associated with the total fat. In addition, eight lipids of TG [(12:1e_6:0_18:0), CL (78:11), TG (17:0_6:0_18:2), SPH(d18:1), PE (16:1e_22:6), PC (17:1_16:0), TG (12:0e_10:3_18:1), and LBPA (16:0_18:1)] were positively associated with CHOL, while only two lipids of MGDG (38:1e) and TG (18:4_18:2_18:3) were negatively associated with CHOL. Conversely, there were only two lipids of DG (22:5_22:5) and TG (12:0e_10:3_18:1) that were positively and eight lipids of TG (15:0_18:2_22:5), ChE (20:4), PC (33:3), BisMePA (18:2_18:2), MePC (30:0), and MGDG (38:1e) that were negatively associated with blood glucose.

Next, the results of correlations between the intestinal flora phyla and pharmacological factors are shown in Figure 11B; the phylum of *Tenericutes* was positively and the phyla of *Verrucomicrobia* and *Planctomycetes* were negatively associated with four pharmacological factors. The phylum of *Patescibacteria* was negatively associated with TG, total fat, and blood glucose, whereas the phylum of *Fusobacteria* was negatively linked to total fat. Then, the results of correlations between the intestinal flora phyla and lipid metabolites revealed that eight phyla exhibited a positive or negative correlation with lipid metabolites. Among these eight phyla, *Firmicutes*, *Verrucomicrobia*, *Tenericutes*, and *Bacteroidetes* exhibited greater association with the lipid metabolites. For example, the phylum of *Firmicutes* was negatively associated with DG (34:2), but positively linked to MePC (30:0), BisMePA (18:2_18:2), PC (33:3), Hex2Cer (d14:1_20:4), MGDG (38:1e), PC (41:3e), PC (36:1e), PC (36:2e), ChE (20:4), and TG (37:6). The phylum of *Verrucomicrobia* was negatively associated with TG (22:5_17:1_18:2), TG (12:0e_10:3_18:1), CL (78:11), DG (34:2), TG (20:4_22:6_22:6), and TG (20:2_20:2_20:5) but positively linked to TG (18:0_18:0_20:4), PC (35:3), MePC (34:4), PE (38:4), MePC (30:0), BisMePA (18:2_18:2), PC (33:3), Hex2Cer (d14:1_20:4), MGDG (38:1e), PC (36:1e), and SM(d18:1_24:0). The phylum of *Tenericutes* was negatively associated with PC (39:6), TG (22:5_17:1_18:2), TG (12:0e_10:3_18:1), CL (78:11), PC (18:2_20:4), DG (34:2), and TG (20:2_20:2_20:5), but positively linked to MePC (34:4), PE (38:4), MePC (30:0), BisMePA (18:2_18:2), PC (33:3), Hex2Cer (d14:1_20:4), MGDG (38:1e), SM (44:4), and PE (17:1_20:4). The phylum of *Bacteroidetes* was positively associated with TG (20:2_20:2_20:5) and positively linked to MePC (30:0), BisMePA (18:2_18:2), PC (33:3), Hex2Cer (d14:1_20:4), PC (41:3e), PC (36:1e), PC (36:2e), and TG (37:6). However, the other four phyla including *Patescibacteria*, *Planctomycetes*, *Acidobacteria*, and *Fusobacteria* displayed less association with the lipid metabolites. For instance, the phylum of *Patescibacteria* was only negatively associated with SM (d18:1_24:0) but positively linked to PC (17:1_16:0), PE (16:1e_22:6), PC (39:6), PC (17:0_22:6), and PC (30:0). On the other hand, the phylum of *Acidobacteria* merely displayed a positive association with SPH (d18:1), PC (17:0_22:6), TG (12:1e_6:0_22:5), and TG (37:6). The phylum of *Fusobacteria* showed a positive association with SPH (d18:1).

Finally, the results of correlations between intestinal flora genera and pharmacological factors and between intestinal flora genera and lipid metabolites (Figure 11C) were analyzed. The results showed numerous associations between intestinal flora genera and pharmacological factors. For example, the genera of *Oscillospira*, *Alloprevotella*, *Weissella*, *Mycoplasma*, *Holdemania*, *Aerococcus*, *Proteus*, and *Shuttlewothia* were positive with four pharmacological factors. Conversely, the genera of *Lachnospiraceae_UCG-006*, *Akkermansia*, *Turicibacter*, *Candidatus_Saccharimonas*, *Bacteroides_pectinophilus_ group*, *Prevotella_9*, *Acetatifactor*, *Ruminococcaceae_UCG-013*, *Negativibacillus*, *GCA-900066575*, *Dubosiella*, and *Romboutsia* were negatively correlated*.* Numerous associations were also observed between the intestinal flora genera and lipid metabolites. For example, *Lactobacillus*, *Prevotellaceae_NK3B31_group*, and *Blautia* were identified as the top three dominant genera in the samples in Section 3.5. Among these three genera, the genus of *Lactobacillus* exhibited a positive correlation with the lipids of MGDG (38:1e), Hex2Cer (d14:1_20:4), PE (17:1_20:4), and PE (38:4) and a negative association with the lipids of DG (34:2), CL (78:11), TG (20:4_22:6_22:6), and TG (20:2_20:2_20:5). On the other hand, the genus of *Prevotellaceae_NK3B31_group* showed significant negative association with the lipids of ChE (20:4), MePC (30:0), BisMePA (18:2_18:2), PC (33:3), MGDG (38:1e), PC (41:3e), PC (36:1e), and PC (36:1e) and a remarkable positive association with the lipids of DG (22:6_22:6), TG (12:0e_10:3_18:1), and DG (34:2). The genus of *Blautia* was positively correlated with the lipids of Hex2Cer (d14:1_20:4), PE (17:1_20:4), and PE (38:4) and negatively associated with CL (78:11) and TG (20:4_22:6_22:6). Collectively, the aforementioned data indicated that the variations in intestinal flora and lipid metabolites were closely linked to each other and both of them were simultaneously associated with the pharmacological factors of total fat, CHOL, TG, and blood glucose in the BYHWD-treated ZDF rats.

## 4 Discussion

In the present study, the first interesting discovery is that BYHWD exhibited hypoglycemic and lipid-lowering effects in diabetic ZDF rats, characterized by the inhibition of body fat accumulation and reduction in blood glucose levels and serum CHOL and TG levels. Our finding is consistent with that of a prior report in which BYHWD could ameliorate serum lipid levels (TC/TG/HDL-C/LDL-C) in the atherosclerotic rat (Liu et al., 2020). Additionally, another research reported that BYHWD alleviated cardiac remodeling-induced pressure overload and exhibited a cardioprotective effect (Chen et al., 2017). Although these studies have demonstrated BYHWD as a TCM recipe, it is well-known as a representative prescription for the treatment of qi-deficiency and blood stasis syndrome symptoms such as ischemic stroke, atherosclerosis, and other cardiovascular diseases. BYHWD is composed of seven herbal medicines, of which Radix Astragali is the dominant herbal medicine because the amount of Radix Astragali in this formula is five-folds higher than the total amount of the other six herbal medicines. Radix Astragali has been studied widely for treating diabetes mellitus, with promising effects observed in recent years. For example, an earlier study revealed that Radix Astragali exhibited the bioactivities of weight loss, improvement in insulin sensitivity, and attenuation of fatty liver in diabetic SD rats (Juan et al., 2011). Another study reported that Radix Astragali improves dysregulated TG metabolism characterized by decreasing fasting TG levels, FFA concentrations, and adipocyte size in HFD-induced obese rats (Long et al., 2014). A recent study also revealed that Radix Astragali promoted fatty acid metabolism and maintained fatty acid homeostasis to alleviate doxorubicin-induced cardiotoxicity in mice (Han et al., 2022). These pharmacological activities such as body weight loss, decrease in TG metabolism, and alleviation of FFA concentrations are consistent with the major pharmacological activities of BYHWD observed in the present study.

The second interesting discovery of the present study is that gut microbiota dysbiosis is closely related to HFD-induced abnormal lipid metabolism in diabetic ZDF rats. BYHWD exhibited unique properties to adjust the abnormal lipid metabolism and meliorate gut microbiota dysbiosis in diabetic ZDF rats. The species richness and diversity of intestinal microbiota are key factors to maintain the relative stability of the intestinal microbiota. Several studies have revealed that the occurrence of T2DM and obesity are closely related to the changes in intestinal microbiota richness and diversity (Graessler et al., 2013; Lambeth et al., 2015). In the present study, for example, at the phylum level, the relative abundances of *Bacteroidetes* and *Proteobacteria* significantly increased, whereas that of *Firmicutes* dramatically decreased in the diabetic ZDF rats after BYHWD treatment. *Bacteroidetes* and *Firmicutes* are the major phyla in the intestinal flora. Jeffrey I. Gordon, a famous scientist in the gut microflora field, revealed that obesity is closely related to changes in the relative abundances of these two dominant bacterial divisions (Turnbaugh et al., 2006). These studies have found that the relative abundance of *Bacteroidetes* in ob/ob mice was lower by 50%, whereas that of *Firmicutes* was higher by a corresponding degree. Some other studies have also reported that the gut microbiota of obese animals and humans exhibited a higher *Firmicutes*/*Bacteroidetes* ratio compared with that of normal weight individuals. Accordingly, the *Firmicutes*/*Bacteroidetes* ratio is frequently cited in most of the scientific literature as a hallmark of obesity (Armougom and Raoult, 2008; Magne et al., 2020). In our present work, the ZDF rats in the DM group also exhibited a significantly higher *Firmicutes*/*Bacteroidetes* ratio of 1.36. However, the ZDF rats in the BYHWD group had a lower *Firmicutes/Bacteroidetes* ratio of 0.78. In addition to alterations in both dominant phyla, BYHWD altered the other phyla, for example, the relative abundances of *Verrucomicrobia* and *Planctomycetes* in the DM group were significantly decreased, while the relative abundance of *Verrucomicrobia* species in the BYHWD group was significantly increased. Studies have found that some bacterial genera in the phylum *Verrucomicrobia* are involved in sulfur metabolism and mucin degradation, which are closely related to lipid metabolism (Derrien et al., 2004). In addition, at the genus level, the BYHWD group exhibited significantly increased relative abundances of *Lactobacillus* and *Blautia* 2.45- and 2.47-fold, respectively, than the DM group. *Lactobacillus* is a probiotic present in mammals that provides critical defense against pathogens and has strong adhesion ability to the intestinal mucosa. It can improve the distribution of intestinal flora, competitively antagonize the colonization of harmful bacteria, enhance the body’s immune function, and has a certain impact on lipid metabolism (Guimarães et al., 2020; Zhou et al., 2021). Many studies have reported that the presence of this bacterium inversely correlates with body weight in rodents and humans (Everard et al., 2013). For example, some studies have found that *Lactobacillus* reduces body weight gain and fat accumulation and lowers the levels of plasma insulin, leptin, total cholesterol, and liver toxicity biomarkers in HFD-induced obese mice (Cani et al., 2008; Park et al., 2013; Crovesy et al., 2017). Moreover, *Blautia* is a genus of anaerobic bacteria with probiotic characteristics that occur widely in the feces and intestines of mammals. Several studies have recently focused on determining its ability to alleviate metabolic syndrome (Liu et al., 2021). For instance, some studies have uncovered *Blautia* as the strongest predictor of weight loss when present in high abundance at the baseline. *Blautia* dramatically restored the proportions of HFD-affected bacteria and raised the abundance of *Blautia*, which is negatively correlated to obesity (Kong et al., 2019; Jie et al., 2021; Reimer and Delzenne, 2021). Moreover, compared with the control group, the abundances of the genera of *Akkermansia* and *Eubacterium* in the DM group were significantly decreased, whereas that of *Alloprevotella* was significantly increased. However, compared with the DM group, the BYHWD group increased the abundances of *Akkermansia*, *Butyricicoccus*, *Parasutterella*, and *Eubacterium*, whereas decreased the abundances of *Alloprevotella* and *Prevotellaceae*. Some studies have found that the relative abundance of *Akkermansia* was negatively correlated to obesity and that it helps improve obesity and insulin resistance (Zhang et al., 2009; Santacruz et al., 2010; Everard et al., 2013). The main metabolite of *Akkermansia* is propionic acid, a short-chain fatty acid (SCFA), which can inhibit cholesterol synthesis in the liver by acting on hydroxymethylglutarate monoacyl-CoA reductase (Markowiak-Kopeć and Śliżewska, 2020). Meanwhile, propionic acid is proven to inhibit appetite, stimulate intestinal gluconeogenesis, promote glucagon-like peptide secretion, improve insulin resistance, and reduce blood glucose levels (Tolhurst et al., 2012; Li et al., 2018). Other studies have also found that *Eubacterium* can produce butyrate and propionate and its nutrient interaction with *Bifidobacterium* promotes SCFA generation (Bunesova et al., 2018). *Parasutterella* can decrease low-density lipoprotein, and *Butyricicoccus* can produce butyrate and decrease inflammatory reactions (Bush and Alfa, 2020). Collectively, in the present study, BYHWD reduced body weight, inhibited fat accumulation, decreased TGs in blood, and improved insulin resistance. This bioactivity of BYHWD is closely associated with the target intestinal flora such as *Firmicutes*, *Bacteroidetes*, *Lactobacillus*, and *Blautia*. Further studies are warranted to investigate the underpinning mechanisms of how these identified intestinal florae affect glycolipid metabolism under BYHWD treatment.

Another interesting finding is the presence of some close associations between liver lipid metabolites and pharmacological factors after BYHWD treatment in diabetic ZDF rats. BYHWD not only significantly decreased blood TG levels in the blood but also notably inhibited the excessive lipid deposition in the liver of ZDF rats under high lipid stress. Liver steatosis is one of the most important clinical and pathological features of obese patients with T2DM, which is characterized by fat accumulation in the form of lipid droplets in the liver cytoplasm (Batista-Gonzalez et al., 2020). TGs are the main lipids contained in lipid droplets, and TGs ingested in food are transported in the form of chylomicrons. Among them, 80% TGs are hydrolyzed to FFAs and absorbed by the adipose tissue, and 20% TGs are ingested by the liver (Titchenell et al., 2017). Because HFD can increase the amount of fat transported to the liver, it is also a common method for inducing hepatic steatosis in animal models (Hariri and Thibault, 2010). In obese patients with T2DM, TGs directly ingested by the liver, the FFAs (hydrolyzed by adipose tissue and then transported to the liver), and *de novo* synthesis of liver fat are all increased, which means fatty acids in hepatocytes are increased (de Souza et al., 2015). Numerous studies have proven that under the conditions of over nutrition and obesity, hepatic lipid metabolism is markedly altered, commonly leading to the amassing of TG in hepatocytes, and this results in a clinical syndrome known as non-alcoholic fatty liver disease (NAFLD) (Alves-Bezerra and Cohen, 2017; Mizuno, 2018). The present study results suggested that BYHWD has a good effect on reducing blood TG and inhibiting liver lipid deposition, which is closely associated with the improvement of the lipid metabolism pattern in the liver. Because the intestinal microflora is a crucial player in obesity and lipid metabolism, as indicated by the present study results, BYHWD upregulated 19 specific lipid metabolites. Some of these metabolites were positively associated with *Bacteroidetes*, *Lactobacillus*, and *Blautia* exhibiting their beneficial function of weight loss and inhibition of fat accumulation in the liver. While BYHWD downregulated 25 lipid metabolites, some of them were positively associated with *Firmicutes* to display effective bioactivity of inhibiting HFD-induced weight and fat gain in diabetic ZDF rats. Dysregulation of the intestinal flora can specifically activate FFAs as nutrient-sensing receptors, leading to an increased flow of FFAs to the liver and promoting *de novo* synthesis of TGs in the liver, resulting in liver lipid degeneration. The study results showed that after BYHWD treatment of T2DM rats, the richness and species diversity of the intestinal flora was recovered and the flora species structure was similar to that in the rats from the control group. BYHWD may regulate the richness and diversity of intestinal microbiota in T2DM rats to restore the species structure of intestinal microbiota. It may also maintain the stability of the intestinal microbiota to play a pharmacodynamic role in regulating liver lipid metabolism. However, the alternations in hepatic lipid metabolic profiles may not only be merely due to the change in the intestinal flora but also due to the change in the expression of genes or proteins. Indeed, additional studies are required to explore the deep mechanistic basis of the bioactivity of BYHWD to regulate lipid abnormalities.

## 5 Conclusion

Our present study results show that BYHWD can significantly inhibit body fat accumulation, reduce the levels of cholesterol and triglycerides, and dramatically lower the blood glucose levels in ZDF rats with obesity and T2DM. The ability of BYHWD to lower blood glucose levels is similar to the effect of metformin, and its ability to reduce serum TG levels was superior to that of metformin. In particular, BYHWD can significantly inhibit the excessive deposition of lipids in the liver of ZDF rats under HFD-induced high lipid stress. More importantly, an integrative analysis method of the gut flora and hepatic lipid metabolomics revealed that the alteration in the gut flora was linked to alterations in hepatic lipid metabolites and both are correlated to the melioration of HFD-induced abnormal blood glucose and serum lipid levels, and obesity in the obese ZDF rats with T2DM. Our findings provide clues to better explore the mechanisms underlying the bioactivity of BYHWD on obesity and T2DM and its application as an effective complementary drug or therapy for the improved treatment of obesity and T2DM.

## Data Availability

The datasets presented in this study can be found in online repositories. The names of the repository/repositories and accession number(s) can be found at: https://www.ncbi.nlm.nih.gov/- PRJNA877465.

## Abbreviation

BisMePA, bis-methyl phosphatidic acid; BYHWD, *Buyang Huanwu* decoction; Cer, ceramides; CL, cardiolipins; CHOL, cholesterol; DG, diglyceride; DMBG, metformin hydrochloride tablets; FBG, fasting blood glucose; HDL, high-density lipoprotein; HOMA-IR, homeostasis model assessment-insulin resistance; IR, insulin resistance; KEGG, Kyoto Encyclopedia of Genes and Genomes; LDL, low-density lipoprotein; MePC, methyl phosphatidylethanolamine; OPLS-DA, orthogonal least squares-discriminant analysis; PC, phosphatidylcholines; PE, phosphatidylethanolamines; PCA, principal component analysis; PLS-DA, partial least squares-discriminant analysis; SM, sphingomyelin; TG, triglyceride; T2DM, type 2 diabetes mellitus; ZDF, Zucker diabetic fatty; ZLC, Zucker lean control.
