# Supplementary material for: Buyang Huanwu decoction affects gut microbiota and lipid metabolism in a ZDF rat model of co-morbid type 2 diabetes mellitus and obesity: An integrated metabolomics analysis
Source: Front Chem. 2022 Nov 9;10:1036380. doi: 10.3389/fchem.2022.1036380 (PMC9682010; doi:10.3389/fchem.2022.1036380)
Supplement: Supplementary file 2 [file DataSheet1.docx]

**Supplementary Materials**

***BuYangHuanWu* Decoction affects the gut microbiota and liver lipid metabolism in a ZDF rat model of co-morbid type 2 diabetes mellitus and obesity: an integrated metabolomics analysis**

Mei Liu^1^, Xinmian Zhao^2^, Jiayan Liu ^2^, Aijing Huang^2^, XinHua Xia ^2,3, *^

*^1^ School of Agriculture and Biology, Zhongkai University of Agriculture and Engineering, No. 501 Zhongkai Rd, Haizhu District, Guangzhou, Guangdong Province 510225, P.R. China.*

*E-mail:* [*maymayliu77@hotmail.com*](mailto:maymayliu77@hotmail.com)<mailto:liumei2007@gzucm.edu.cn>*.*

*^2^ The First Affiliated Hospital of Guangzhou Medical University, No. 151 West Yanjiang Road, Yuexiu District, Guangzhou, Guangdong Province 510120, P.R.China.. E-mail:* [*790001034@qq.com*](mailto:790001034@qq.com)*,* [*823939799@qq.com*](mailto:823939799@qq.com)*.*

*^3^ Institute of integrated Chinese and Western Medicine, Guangzhou Medical University, No.195 West Dongfeng Road, Yuexiu Distric, Guangzhou, Guangdong Province 510182, P.R.China. E-mail: star1124@163.com*

*Corresponding author: Xin-hua Xia, Ph.D, Professor, *No. 151 West Yanjiang Road, Yuexiu District, Guangzhou, Guangdong Province 510120, P.R.China.*Tel.: 86-020-89003030; Fax: 86-020-81377207. Email: [star1124@163.com](mailto:star1124@163.com)


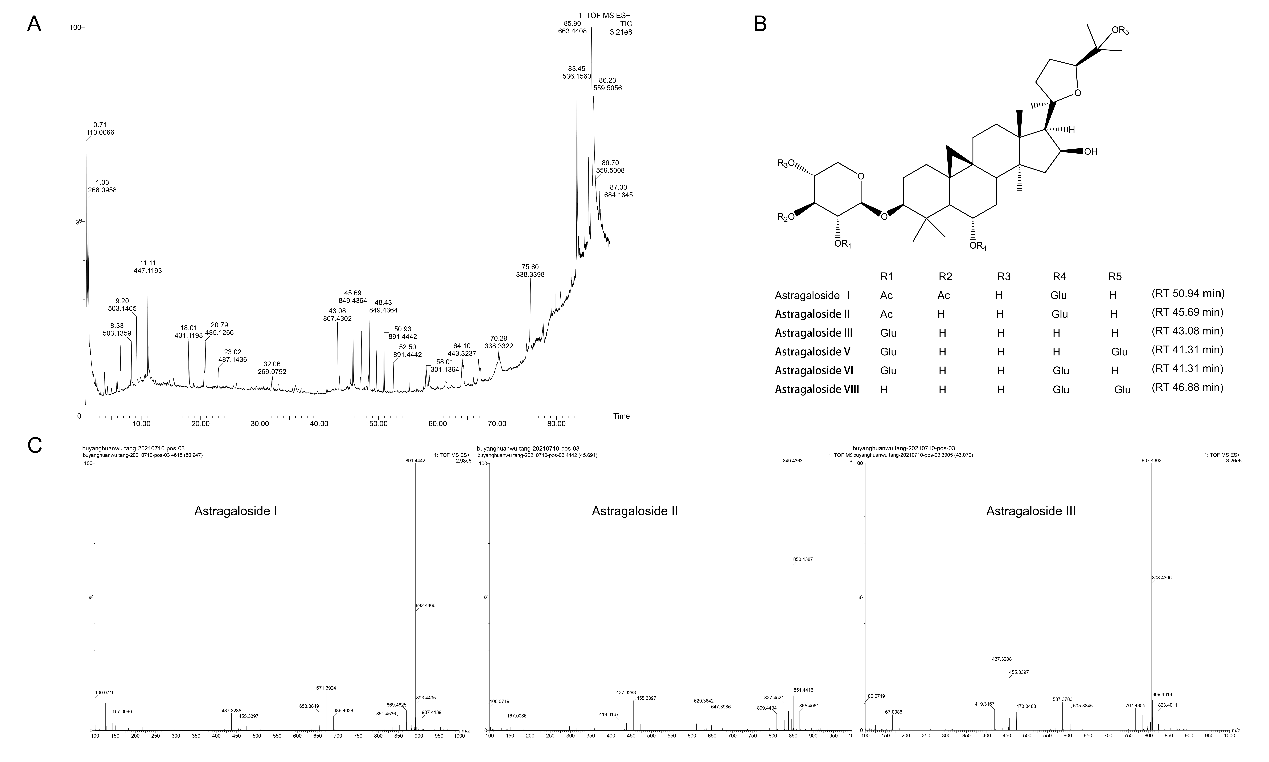


Supplementary Figure 1. The images of characteristics components extracted from BYHWD determined by LC-QTOF-MS: (A), The TIC chromatogram of BYHWD water extract; (B), the representative structures of Astragaloside I, II, III, IV, V mainly extracted from Astragali Radix that is the major herbal medicine of BYHWD; (C), the representative image of MS fragment of Astragaloside I, II, III.


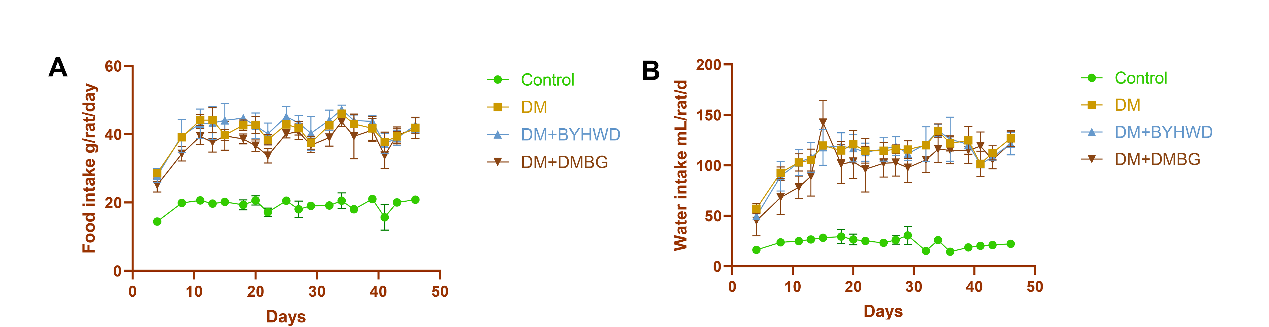


Supplementary Figure 2. BYHWD attenuated the body weight increase and fat accumulation induced in HFD: (A), Average food intake during treatments; (B), Average water intake during treatments.

Supplement-table 1. The LC-MS characteristics of seventy-seven identified components from BYHWD

| No. | Component name | Observed RT  (min) | Formula | Neutral mass (Da) | Observed m/z | Response | Adducts | Resources | Cite |
| --- | --- | --- | --- | --- | --- | --- | --- | --- | --- |
| 1 | Nicotinic acid | 1.00 | C_6_H_5_NO_2_ | 123.03203 | 124.0393 | 80253 | +H | Angelicae sinensis radix | (Liu et al., 2009) |
| 2 | Gallic acid | 1.41 | C_7_H_6_O_5_ | 170.02152 | 171.0285 | 46596 | +H | Paeoniae Rubra Radix | (Tan et al., 2020) |
| 3 | Phenylpropionic acid | 2.02 | C_9_H_11_NO_2_ | 165.07898 | 166.086 | 269665 | +H, +Na | Astragali Radix | (Qi et al., 2009) |
| 4 | Almond cyanide | 2.53 | C_8_H_7_NO | 133.05276 | 134.0597 | 28952 | +H | Persicae Semen | (Yang et al., 2014) |
| 5 | Lactinolide | 3.33 | C_10_H_16_O_4_ | 200.10486 | 223.0936 | 16566 | +Na | Paeoniae Rubra Radix | (Xu et al., 2009) |
| 6 | 6-O-β-D-Glucopyranosyl lactinolide | 3.39 | C_16_H_26_O_9_ | 362.15768 | 385.1467 | 24027 | +Na | Paeoniae Rubra Radix | (Xu et al., 2009) |
| 7 | Oxypaeoniflora | 4.9 | C_23_H_28_O_12_ | 496.15808 | 519.1477 | 23160 | +Na, H | Paeoniae Rubra Radix | (Lu et al., 2015) |
| 8 | Quercetin-3-methylether | 4.98 | C_16_H_12_O_7_ | 316.0583 | 339.0466 | 11597 | +Na | Persicae Semen | (Horton et al., 2002) |
| 9 | Hydroxylsafflower yellow | 5.96 | C_27_H_32_O_16_ | 612.16903 | 635.1582 | 214725 | +Na, H | Persicae Semen | (Yang et al., 2020) |
| 10 | 3-methoxy-4-hydroxyl-Benzoic acid | 6.12 | C8H8O4 | 168.04226 | 191.0315 | 18077 | +Na | Chuanxiong Rhizoma | (Wang et al., 2020) |
| 11 | 7-hydroxy--3',4'-dimethoxy-2’5’-dicosaccharide-isoflavane | 7.48 | C29H38O16 | 642.21599 | 665.2052 | 15581 | +Na | Astragali Radix | (Wang et al., 2008) |
| 12 | Anisic acid | 7.77 | C8H8O3 | 152.04734 | 153.0542 | 10392 | +H | Angelicae sinensis radix | (Yi et al., 2009) |
| 13 | Prunasin | 7.78 | C_14_H_17_NO_6_ | 295.10559 | 318.0948 | 20606 | +Na | Persicae Semen  Carthamiflos | (Yang et al., 2014; Liu et al., 2014) |
| 14 | 8-Debenzoylpaeoniflorin | 8.06 | C_16_H_24_O_10_ | 376.13695 | 377.1453 | 38225 | +H,+Na | Paeoniae Rubra Radix | (Copps and White, 2012) |
| 15 | Riboflavin | 8.06 | C_17_H_20_N_4_O_6_ | 376.13828 | 377.1453 | 38225 | +H,+Na | Carthamiflos | (Davis, 2004) |
| 16 | Paeonilactone B | 8.33 | C_10_H_12_O_4_ | 196.07356 | 197.0805 | 57534 | +H,+Na | Paeoniae Rubra Radix | (Lu et al., 2015) |
| 17 | Paeonilactone C | 8.33 | C_17_H_18_O_6_ | 318.11034 | 319.1175 | 97501 | +H, Na | Paeoniae Rubra Radix | (Lu et al., 2015) |
| 18 | Albiflorin | 8.34 | C_23_H_28_O_11_ | 480.16316 | 503.1526 | 1715346 | +Na,+H | Paeoniae Rubra Radix | (Ren et al., 2009) |
| 19 | Albiflorin R1 | 9.21 | C_23_H_28_O_11_ | 480.16316 | 503.1527 | 3370211 | +Na,+H | Paeoniae Rubra Radix | (Ren et al., 2009) |
| 20 | Ferulic Acid | 9.88 | C_10_H_10_O_4_ | 194.05791 | 195.0647 | 46495 | +H | Chuanxiong Rhizoma Angelicae sinensis radix | (Liu et al., 2014)  (Liu et al., 2009) |
| 21 | 3'-methoxy-5'-hydroxy-isoflavone-7-O-β-D-glucoside | 11.12 | C_22_H_22_O_10_ | 446.1213 | 447.1286 | 2817895 | +H,+Na | Astragali Radix | (Wang et al., 2008) |
| 22 | D-catechin | 11.7 | C_15_H_14_O_6_ | 290.07904 | 291.0863 | 11270 | +H | Chuanxiong Rhizoma | (Wang et al., 2020) |
| 23 | Apigenol | 11.75 | C_15_H_10_O_5_ | 270.05282 | 271.0603 | 18753 | +H | Pheretima | (Cheng et al., 2020) |
| 24 | Galloyl-paeoniflorin | 12.11 | C_30_H_32_O_15_ | 632.17412 | 655.1629 | 166027 | +Na | Paeoniae Rubra Radix | (Liu et al., 2014) |
| 25 | Kaempferol-4-methylether-3-glucoside | 14.42 | C_22_H_22_O_11_ | 462.11621 | 463.1237 | 156744 | +H,+Na | Carthamiflos | (Li et al., 2015) |
| 26 | 3',4'-dimethoxy-7,2’- dicosaccharide-astragaloside | 15.37 | C_29_H_38_O_15_ | 626.22107 | 649.2096 | 158364 | +Na | Astragali Radix | (Huang et al., 2009) |
| 27 | Lactiflorin | 16.71 | C_23_H_26_O_10_ | 462.1526 | 485.1416 | 41801 | +Na | Paeoniae Rubra Radix | (Yang et al., 2020) |
| 28 | Formononetin | 18.01 | C_16_H_12_O_4_ | 268.07356 | 269.0809 | 1372145 | +H, Na | Astragali Radix | (Gampe et al., 2016) |
| 29 | Benzoyloxypaeoniflorin | 19.03 | C_30_H_32_O_13_ | 600.18429 | 623.1738 | 7491 | +Na | Paeoniae Rubra Radix | (Lu et al., 2015) |
| 30 | Calycosin | 20.62 | C_16_H_12_O_5_ | 284.06847 | 285.0757 | 1905742 | +H,+Na | Astragali Radix | (Yin et al., 2018) |
| 31 | Evofolin B | 20.65 | C_17_H_18_O_6_ | 318.11034 | 319.117 | 32383 | +H | Paeoniae Rubra Radix | (Wang et al., 2005) |
| 32 | (6αR, 11αR)-10-hydroxy-3,9-dimethoxypterocarpan | 20.79 | C_17_H_16_O_5_ | 300.09977 | 301.1071 | 1802408 | +H | Astragali Radix | (Yin et al., 2018) |
| 33 | 9,10-dimethoxy-pterocarpane-3-O-β-D-glucoside | 20.79 | C_23_H_26_O_10_ | 462.1526 | 485.1422 | 3068707 | +Na,+H | Astragali Radix | (Yin et al., 2018) |
| 34 | Paeonol | 20.79 | C_9_H_10_O_3_ | 166.06299 | 167.0703 | 309253 | +H | Paeoniae Rubra Radix | (Krawczyk et al., 2010) |
| 35 | 2’-hydroxy-3,4-dimethoxyphenyl-isoflavan-7-β-D-glucopyranoside | 23.01 | C_23_H_28_O_10_ | 464.16825 | 487.158 | 1662571 | +Na, H | Astragali Radix | (Yin et al., 2018) |
| 36 | 8,2'-dihydroxy-7,4'-dimethoxy-isoflavan | 23.01 | C_17_H_18_O_5_ | 302.11542 | 303.1228 | 286287 | +H, Na | Astragali Radix | (Bie et al., 2006) |
| 37 | 2′-hydroxy-7,3′,4′-Trimethoxyisoflavan | 23.03 | C_18_H_20_O_5_ | 316.13107 | 339.1207 | 4447 | +Na | Astragali Radix | (Huang et al., 2009) |
| 38 | Odoratin-7-O-β-D-glucopyranoside | 23.88 | C_22_H_24_O_9_ | 432.14203 | 455.1312 | 18348 | +Na | Astragali Radix | (Huang et al., 2009) |
| 39 | Sebacic acid | 24.99 | C_10_H1_8_O_4_ | 202.12051 | 225.1091 | 15696 | +Na | Angelicae sinensis radix | (Li et al., 2020) |
| 40 | Kaempferide | 27.67 | C_16_H_12_O_6_ | 300.06339 | 301.0721 | 55230 | +H, Na | Carthamiflos | (Li et al., 2015) |
| 41 | Benzoylpaeoniflorin | 28.64 | C_30_H_32_O_12_ | 584.18938 | 607.1786 | 253055 | +Na | Paeoniae Rubra Radix | (Lu et al., 2015) |
| 42 | Z-Ligustilide | 30.34 | C_12_H_14_O_2_ | 190.09938 | 191.1064 | 86873 | +H | Chuanxiong Rhizoma Angelicae sinensis radix | (Wang et al., 2020)  (Yi et al., 2009) |
| 43 | 7,2′-dihydroxy-3′,4′-dimethoxy-isoflavan | 35.96 | C_17_H_18_O_5_ | 302.11542 | 303.1228 | 275793 | +H | Astragali Radix | (Huang et al., 2009) |
| 44 | 8,2′-dihydroxy-7,4′-dimethoxy-isoflavan | 35.96 | C_17_H_18_O_5_ | 302.11542 | 303.1228 | 275793 | +H | Astragali Radix | (Huang et al., 2009) |
| 45 | Benzoic acid | 35.96 | C_7_H_6_O_2_ | 122.03678 | 123.0435 | 102938 | +H | Paeoniae Rubra Radix | (Wang et al., 2005) |
| 46 | Astramembrannin C | 39.71 | C_36_H_60_O_10_ | 652.41865 | 675.4072 | 78885 | +Na | Astragali Radix | (Huang et al., 2009) |
| 47 | Senkyunolide | 40.94 | C_12_H_16_O_2_ | 192.11503 | 215.1039 | 56028 | +Na,+H | Angelicae sinensis radix Chuanxiong Rhizoma | (Liu et al., 2009)  (Liu et al., 2014) |
| 48 | Astragaloside Ⅴ | 41.31 | C_47_H_78_O_19_ | 946.51373 | 969.5024 | 327490 | +Na,+H | Astragali Radix | (Qi et al., 2009) |
| 49 | Astragaloside Ⅵ | 41.31 | C_47_H_78_O_19_ | 946.51373 | 969.5024 | 327490 | +Na, H | Astragali Radix | (Qi et al., 2009) |
| 50 | Oplopanaxogenin C | 43.08 | C_30_H_48_O_4_ | 472.35526 | 473.3629 | 207512 | +H | Astragali Radix | (Gampe et al., 2016) |
| 51 | Astragaloside Ⅲ | 43.08 | C_41_H_68_O_14_ | 784.46091 | 807.4504 | 3203808 | +Na,+H | Astragali Radix | (Zheng et al., 2019) |
| 52 | Isoastragaloside IV | 43.4 | C_41_H_68_O_14_ | 784.46091 | 807.4497 | 593551 | +Na, H | Astragali Radix | (Huang et al., 2009) |
| 53 | Agroastragaloside Ⅳ | 44.24 | C_49_H_80_O_20_ | 988.52429 | 989.5297 | 65218 | +H | Astragali Radix | (Huang et al., 2009) |
| 54 | Astramembrannin B | 45.32 | C_36_H_60_O_10_ | 652.41865 | 675.4073 | 69606 | +Na,+H | Astragali Radix | (Huang et al., 2009) |
| 55 | Astramembrannin II | 45.35 | C_35_H_58_O_9_ | 622.40808 | 645.397 | 316993 | +Na | Astragali Radix | (Qi et al., 2009) |
| 56 | Astragaloside Ⅱ | 45.70 | C_43_H_70_O_15_ | 826.47147 | 849.4606 | 3220592 | +Na,+H | Astragali Radix | (Zheng et al., 2019) |
| 57 | 3,4- dimethoxy benzaldehyde | 46.07 | C_9_H_10_O_3_ | 166.06299 | 167.0707 | 47977 | +H | Paeoniae Rubra Radix | (Liu et al., 2009) |
| 58 | Astragaloside Ⅷ | 46.88 | C_47_H_76_O_17_ | 912.50825 | 935.496 | 68438 | +Na,+H | Astragali Radix | (Zheng et al., 2019) |
| 59 | Astragaloside Ⅱ | 47.12 | C_43_H_70_O_15_ | 826.47147 | 849.4602 | 2129201 | +Na,+H | Astragali Radix | (Zheng et al., 2019) |
| 60 | Astragaline E | 48.05 | C_14_H_16_N_2_O_5_ | 292.10592 | 293.1144 | 11286 | +H | Astragali Radix | (Gampe et al., 2016) |
| 61 | Astragaloside Ⅱ | 48.44 | C_43_H_70_O_15_ | 826.47147 | 849.4604 | 2499526 | +Na, H | Astragali Radix | (Zheng et al., 2019) |
| 62 | Mongholicoside Ⅰ | 50.85 | C_36_H_60_O_9_ | 636.42373 | 659.4117 | 6747 | +Na | Astragali Radix | (Gampe et al., 2016) |
| 63 | Agroastragaloside Ⅱ | 50.94 | C_43_H_72_O_15_ | 828.48712 | 851.4772 | 215669 | +Na | Astragali Radix | (Yin et al., 2018) |
| 64 | Astragaloside Ⅰ | 50.94 | C_45_H_72_O_16_ | 868.48204 | 891.4695 | 2403609 | +Na,+H | Astragali Radix | (Zheng et al., 2019) |
| 65 | Linolenic acid | 50.96 | C_18_H_30_O_2_ | 278.22458 | 279.2312 | 13377 | +H | Astragali Radix | (Yin et al., 2018) |
| 66 | Acetytastragaloside | 55.16 | C_47_H_74_O_17_ | 910.4926 | 933.4802 | 610075 | +Na, H | Astragali Radix | (Yin et al., 2018) |
| 67 | Polycanthine | 56.49 | C_15_H_19_NO_3_ | 261.13649 | 262.1433 | 53859 | +H | Astragali Radix | (Gampe et al., 2016) |
| 68 | Phthalic anhydride | 58.03 | C_8_H_4_O_3_ | 148.01604 | 149.0228 | 34394 | +H | Angelicae sinensis radix | (Krawczyk et al., 2010) |
| 69 | Isoeugenol | 58.54 | C_10_H_12_O_2_ | 164.08373 | 165.0906 | 65779 | +H, Na | Paeoniae Rubra Radix | (Huang et al., 2006) |
| 70 | Linolic Acid | 60.12 | C_18_H_32_O_2_ | 280.24023 | 281.2469 | 11521 | +H | Persicae Semen | (Yang et al., 2014) |
| 71 | Palbinone | 64.77 | C_22_H_30_O_4_ | 358.21441 | 381.2053 | 12874 | +Na | Paeoniae Rubra Radix | (Lu et al., 2015) |
| 72 | Ligustilide dimer | 67.87 | C_24_H_28_O_4_ | 380.19876 | 381.2058 | 58372 | +H | Chuanxiong Rhizoma | (Wang et al., 2020) |
| 73 | Palmitic acid | 70.3 | C_16_H_32_O_2_ | 256.24023 | 257.2465 | 6469 | +H | Carthamiflos | (Wang et al., 2008) |
| 74 | Tetradecanoic acid | 72.2 | C_15_H_30_O_2_ | 242.22458 | 243.2314 | 7035 | +H | Carthamiflos | (Wang et al., 2008) |
| 75 | Stearic acid | 74.99 | C_18_H_36_O_2_ | 284.27153 | 285.2788 | 13383 | +H | Persicae Semen | (Yang et al., 2014) |
| 76 | Paeonilactinone | 80.37 | C_10_H_16_O_2_ | 168.11503 | 191.1039 | 20314 | +Na | Paeoniae Rubra Radix | (Lu et al., 2015) |
| 77 | Mongholicoside Ⅱ | 82.99 | C_38_H_62_O_11_ | 694.42921 | 717.4177 | 23008 | +Na | Astragali Radix | (Gampe et al., 2016) |

References

Bie, Y.Y., Guan, J., Bi, Z.M., Song, Y.M., Li, P., 2006. Studies on Chemical Constituents of Astragalus membranaceus (Fisch.) Bge. var. mongholicus (Bge.) Hsiao. Chinese Pharmaceutical Journal 41,1217-1221.

Cheng, T. F., Zhang, Y. H., Ye, J., Jin, H. Z., Zhang, W.D., 2020. Investigation of the chemical compounds in Pheretima aspergillum (E. Perrier) using a combination of mass spectral molecular networking and unsupervised substructure annotation topic modeling together with in silico fragmentation prediction. J. Pharm. Biomed. Anal. 184, 113197.

Fukuda, T., Ito, H., Mukainaka, T., Tokuda, H., Nishino, H., Yoshida, T., 2003. Anti-tumor promoting effect of glycosides from Prunus persica seeds. Biol. Pharm. Bull. 26(2), 271–273. https://doi.org/10.1248/bpb.26.271

Gampe, N., Darcsi, A., Lohner, S., Béni, S., Kursinszki, L., 2016. Characterization and identification of isoflavonoid glycosides in the root of Spiny restharrow (Ononis spinosa L.) by HPLC-QTOF-MS, HPLC-MS/MS and NMR. J. Pharm. Biomed. Anal. 123, 74-81.

He, C., Xiao, W., Li, M., Peng, Y., Xu, L., Gu, J., Xiao, P., 2010. Chemical constituents from seeds of Paeonia suffruticosa. China journal of Chinese materia medica 35(11), 1428–1431.

Huang, W.Y., Sheu, S.J., 2006. Separation and identification of the organic acids in Angelicae Radix and Ligustici Rhizoma by HPLC and CE. J. Separat. Sci. 29(17), 2616–2624.

Huang, X., Liu, Y., Song, F., Liu, Z., Liu, S., 2009. Studies on principal components and antioxidant activity of different Radix Astragali samples using high-performance liquid chromatography/electrospray ionization multiple-stage tandem mass spectrometry. Talanta 78(3), 1090–1101.

Li, X., Yao, Y., Wang, X., An, C., Gao, S., Xiang, F., Dong, Y., 2020. Quantification Analysis of 13 Organic Components and 8 Inorganic Elements in Angelica Sinensis Radix and Its Different Parts Combined with Chemical Recognition Pattern. J. Anal. Methods Chem. 2020, 8836184.

Li, Y., Piao, D., Zhang, H., Kim, T., Lee, S. H., Chang, H. W., Woo, M.H., Son, J.K., 2015. Quality evaluation of Carthami Flos by HPLC-UV. Arch. Pharm. Res. 38(5), 776–784.

Lin, L., Xu, M., Ma, L., Zeng, J., Zhang, F., Qiao, Y., Wu, Z., 2020. A rapid analysis method of safflower (Carthamus tinctorius L.) using combination of computer vision and near-infrared. Mol. Biomol. Spectroscopy 236, 118360.

Liu, E. H., Qi, L. W., Li, B., Peng, Y. B., Li, P., Li, C. Y., Cao, J., 2009. High-speed separation and characterization of major constituents in Radix Paeoniae Rubra by fast high-performance liquid chromatography coupled with diode-array detection and time-of-flight mass spectrometry. Rapid Commun. Mass 23(1), 119–130.

Liu, J.L., Zheng, S. L., Fan, Q. J., Yuan, J. C., Yang, S. M., Kong, F.L., 2014. Optimization of high-pressure ultrasonic-assisted simultaneous extraction of six major constituents from Ligusticum chuanxiong rhizome using response surface methodology. Molecules 19(2), 1887-1911.

Lu, X.H., Ma, X., Wang, J., Zhu, Y., Zhou, Z.Y., Chen, Z., Zhao, Y.L., Wang, J.B., 2015. Research progress on chemical constituents of Paeoniae Rubra Radix and their pharmacological effects. Chinese Traditional and Herbal Drugs 46(4), 595-602.

Qi, L. W., Li, P., Ren, M.T., Yu, Q.T., Wen, X.D., Wang, Y.X., 2009. Application of high-performance liquid chromatography-electrospray ionization time-of-flight mass spectrometry for analysis and quality control of Radix Astragali and its preparations. J. Chromatogr. A 1216(11), 2087–2097.

Ren, M.L., Zhang, X., Ding, R., Dai, Y., Tu, F.J., Cheng, Y.Y., Yao, X.S., 2009. Two new monoterpene glucosides from Paeonia lactiflora Pall. J. Asian Natl Prod. Res. 11(7), 670–674.

Tan, Y.Q., Chen, H. W., Li, J., Wu, Q.J., 2020. Efficacy, Chemical Constituents, and Pharmacological Actions of Radix Paeoniae Rubra and Radix Paeoniae Alba. Front. Pharmacol. 11, 1054.

Wang, L., Huang, S., Chen, B., Zang, X. Y., Su, D., Liang, J., Xu, F., Liu, G. X., Shang, M.Y., Cai, S.Q., 2016. Characterization of the Anticoagulative Constituents of Angelicae Sinensis Radix and Their Metabolites in Rats by HPLC-DAD-ESI-IT-TOF-MSn. Planta Medica 82(4), 362–370.

Wang, R.J., Yang, B., Fu, M.H., 2008.  Quality evaluation of Flos Carthami. China journal of Chinese materia medica, 33(22), 2642–2646.

Wang, X., Cheng, C., Sun, Q., Li, F., Liu, J., Zheng, C., 2005. Isolation and purification of four flavonoid constituents from the flowers of Paeonia suffruticosa by high-speed counter-current chromatography. J. Chromatogr. A 1075(1-2), 127–131.

Wang, X., Yao, Y., An, C., Li, X., Xiang, F., Dong, Y., Li, M., 2020. Simultaneous determination of 20 bioactive components in Chuanxiong Rhizoma from different production origins in Sichuan province by ultra-high-performance liquid chromatography coupled with triple quadrupole mass spectrometry combined with multivariate statistical analysis. Electrophoresis 41(18-19), 1606–1616.

Xu, S., Yang, L., Tian, R., Wang, Z., Liu, Z., Xie, P., Feng, Q., 2009. Species differentiation and quality assessment of Radix Paeoniae Rubra (Chi-shao) by means of high-performance liquid chromatographic fingerprint. J. Chromatogr. A 1216(11), 2163–2168.

Yang, C., Zhao, J., Cheng, Y., Li, X., Rong, J., 2014. Bioactivity-guided fractionation identifies amygdalin as a potent neurotrophic agent from herbal medicine Semen Persicae extract. BioMed Res. Int. 2014, 306857. https://doi.org/10.1155/2014/306857

Yang, Y., Li, S.S., Teixeira da Silva, J. A., Yu, X.N., Wang, L.S., 2020. Characterization of phytochemicals in the roots of wild herbaceous peonies from China and screening for medicinal resources. Phytochemistry 174, 112331.

Yi, L., Liang, Y., Wu, H., Yuan, D., 2009. The analysis of Radix Angelicae Sinensis (Danggui). J. Chromatogr. A 1216(11), 1991–2001.

Yin, G., Cheng, X., Tao, W., Dong, Y., Bian, Y., Zang, W., Tang, D., 2018. Comparative analysis of multiple representative components in the herb pair Astragali Radix-Curcumae Rhizoma and its single herbs by UPLC-QQQ-MS. J. Pharm. Biomed. Anal. 148, 224–229.

Zheng, Y., Duan, W., Sun, J., Zhao, C., Cheng, Q., Li, C., Peng, G., 2019. Structural Identification and Conversion Analysis of Malonyl Isoflavonoid Glycosides in Astragali Radix by HPLC Coupled with ESI-Q TOF/MS. Molecules 24(21), 3929.
